# Atheroprotective Effects and Mechanisms of Postmarketing Chinese Patent Formulas in Atherosclerosis Models: A Systematic Review

**DOI:** 10.1155/2021/4010607

**Published:** 2021-11-27

**Authors:** Shiqi Chen, Xiaoxiao Wu, Tong Li, Yang Li, Baofu Wang, Weiting Cheng, Yu Teng, Jingjing Yang, Hui Meng, Lei Wang, Ziwen Lu, Yangyang Jiang, Yahong Wang, Mingjing Zhao

**Affiliations:** ^1^Key Laboratory of Chinese Medicine of Ministry of Education and Beijing, Dongzhimen Hospital Affiliated to Beijing University of Chinese Medicine, Beijing 100700, China; ^2^Department of Cardiology, Dongzhimen Hospital Affiliated to Beijing University of Chinese Medicine, Beijing 100700, China

## Abstract

**Background:**

Some postmarketing Chinese patent formulas have been widely used to treat atherosclerosis (AS) and play critical roles in Chinese healthcare. However, the usage of these herbs is yet controversial due to unclear effects and lack of understanding of the mechanism of action. With the modernization of traditional Chinese formulas, we are to elucidate the atheroprotective properties of these remedies from successful postmarketing experiments *in vivo*.

**Methods:**

In this systematic review, we critically searched the databases, applied stringent criteria, assessed the methodological quality, and examined the current evidence *in vivo*.

**Results:**

Consequently, 60 studies were included in the present qualitative synthesis. Data on models, high-fat diet, intervention time, outcome measures, efficacy, and mechanisms were collected. Finally, 23 formulas that could alleviate AS were correlated to the amelioration of plaques, improvement of plaque stability, modification of lipid level and lipid metabolism, and the effects of anti-inflammation and antioxidant stress with multiple components and targets. However, the methodological quality was low and incomplete among the included literature.

**Conclusions:**

Thus, taken together, the studies on postmarketing Chinese patent formulas would provide a novel approach to improve the treatment of AS, and rigorously designed studies would provide high-quality evidence.

## 1. Introduction

Cardiovascular diseases (CVDs) are the leading causes of death worldwide, with an estimated 17.9 million deaths each year and 32% of all deaths globally [1, 2]. Compared with the high-income countries (HICs), the death rate of low- and middle-income countries is about three times higher, causing a heavy economic burden to the society [3]. Coronary heart disease (CHD) is one of the CVDs caused by atherosclerosis (AS) in blood vessels and a significant cause of death in both developing and developed countries [4–6]. AS is a multifaceted disorder involving the core mechanisms of endothelium dysfunction, lipid deposition, vascular inflammation, oxidative stress, foam cell formation, and smooth muscle cell migration [7]. In addition, AS can also be identified as an inflammatory disease characterized by immune responses [8]. Modern therapies have made significant progress in the treatment of AS; for example, the Western medicine of statins and the surgeries of percutaneous transluminal coronary intervention (PCI) or coronary artery bypass grafting (CABG). However, interventional therapy can only improve local vascular problems, and statins have been identified to cause side effects of liver damage, muscle dissolution, and new-onset type 2 diabetes mellitus [9]. Therefore, other remedies for improving the efficacy and the adverse reactions are urgent requirements.

Traditional Chinese medicine (TCM) has a history of over 2000 years and has been widely used in Chinese healthcare [10]. Unlike Western medicines, traditional Chinese herbs usually consist of various compounds and provide multiple targets for the prevention and treatment of AS [11]. With widespread clinical applications and adequate tolerance, some TCMs have been deemed as an effective approach for treating ASCVD and restoring the balance of the human body [12].

Hitherto, many Chinese patent formulas produced by pharmaceutical companies have emerged and been approved in China. Several postmarketing studies *in vivo* have been identified to understand the effects and mechanisms of Chinese patent formulas with antiatherosclerotic properties, and some reviews reported the use of Chinese patent formulas in AS [3, 13, 14]. However, most of these reviews were focused on a single formula. In this study, we critically evaluated the effects and summarized the mechanisms of all the postmarketing Chinese patent formulas in treating AS. A systematic review method was implemented by searching the databases, applying strict criteria, assessing the methodological quality, and evaluating outcomes. In this review, we focused on the formulas in the field of AS and the animal models and mechanisms in atheroprotective effects.

## 2. Material and Methods

### 2.1. Information Sources and Search Strategy

The search was applied to three databases, including PubMed, Embase, and Web of Science (publication duration was from the inception through March 29, 2021). The search strategy used the following general terms as mesh terms or free terms: “Medicine, Chinese Traditional”, “Herbal Medicine”, “Drugs, Chinese Herbal”, “Atherosclerosis”, “Chinese patent medicine”, “Chinese patent drug”, “Chinese traditional patent medicine”, “traditional Chinese medicine or Chinese medicine”, “TCM”, “Chinese herb medicine”, “Chinese herbal medicine”, “atherosclerosis”, “arteriosclerosis”, “atherosis”. For instance, the detailed search strategy of PubMed is as follows: (((Medicine, Chinese Traditional) OR (Herbal Medicine) OR (Drugs, Chinese Herbal)) AND (Atherosclerosis)) OR (((Chinese patent medicine or Chinese patent drug or Chinese traditional patent medicine) OR (traditional Chinese medicine or Chinese medicine or TCM or Chinese herb medicine or Chinese herbal medicine)) AND (atherosclerosis or arteriosclerosis or atherosis)). Also, we used a search filter previously developed for PubMed in order to identify all the publications on animal studies [15].

### 2.2. Inclusion and Exclusion Criteria

Considering the objective of our review, the inclusion criteria were listed as follows: (1) original studies were mainly related to the postmarketing Chinese patent formulas *in vivo*; (2) the patent drugs that were approved to treat the related AS diseases could be searched on the website (http://app1.nmpa.gov.cn/data_nmpa/face3/base.jsp?tableId=25&tableName=TABLE25&title=%E5%9B%BD%E4%BA%A7%E8%8D%AF%E5%93%81&bcId=152904713761213296322795806604&CbSlDlH0=qGqJcGqBhnZBhnZBhCJzH3eKIwCWYj2zliOBDl.Z9naqqcZ) of the National Medical Products Administration (NMPA) of the Chinese government; (3) any validated AS model could be applied in this review, including ApoE^−/−^ mice, LDLR^−/−^ mice, New Zealand white rabbits, Japanese white rabbits, Wistar rats, Sprague–Dawley (SD) rats, and C57BL/6 mice with or without a high-fat diet (HFD), balloon injury, and ligation or silastic collar implantation around a specific artery [16, 17]; and (4) language was not restricted, but the literature should have been published in official journals.

The studies were excluded if they were (1) only *in vitro* or clinical research; (2) mainly Chinese medicine monomers or other unlisted formulas; or (3) AS models combined with models of other diseases.

### 2.3. Study Selection and Data Extraction

Two investigators (SQC and XXW) individually conducted the literature search using the predetermined criteria in Endnote X8 software. First, duplicates were identified in various databases and removed from the initial search results. Second, the obviously irrelevant studies were eliminated after reading the titles and abstracts. Third, the full texts were screened to identify the relevant studies, and the unqualified studies were removed. The study selection was independently cross-checked by two researchers. Any disagreement was resolved by discussion in a consensus meeting with the corresponding authors (YHW and MJZ).

Subsequently, two authors (TL and YL) independently extracted data from the included literature employing a standardized sheet prepared for this review, which was further checked by BFW. The extracted data included study title, year of the research publication, drug name, approval number by NMPA, pharmaceutical company, main Chinese herbs, experimental models, HFD feeding time, drug intervention time, outcome measures, efficacy, and mechanisms.

### 2.4. Assessment of Risk of Bias (ROB) in Individual Studies

Two authors (WTC and YT) independently assessed the ROB using SYRCLE's ROB tool [18] for animal studies to evaluate the methodological quality of the included studies with respect to sequence generation (i.e., selection bias), baseline characteristics (i.e., selection bias), allocation concealment (i.e., selection bias), random housing (i.e., performance bias), blinding (i.e., performance bias), random outcome assessment (i.e., detection bias), blinding (i.e., detection bias), incomplete outcomes data (i.e., attrition bias), selective outcome reporting (i.e., reporting bias), and other sources of bias; it was further checked by JJY. The disagreements were resolved by consensus with the corresponding authors (YHW and MJZ).

### 2.5. Summary Measures and Analysis

Regarding the high heterogeneity of the various formulas and the different methodologies, all the outcome measures, which compared the experimental groups with the model groups, were recorded as a significant uptrend marker “↑” or a significant downtrend marker “↓”. The summary analysis was presented using a qualitative synthesis.

## 3. Results

### 3.1. Study Selection

**Figure 1:**
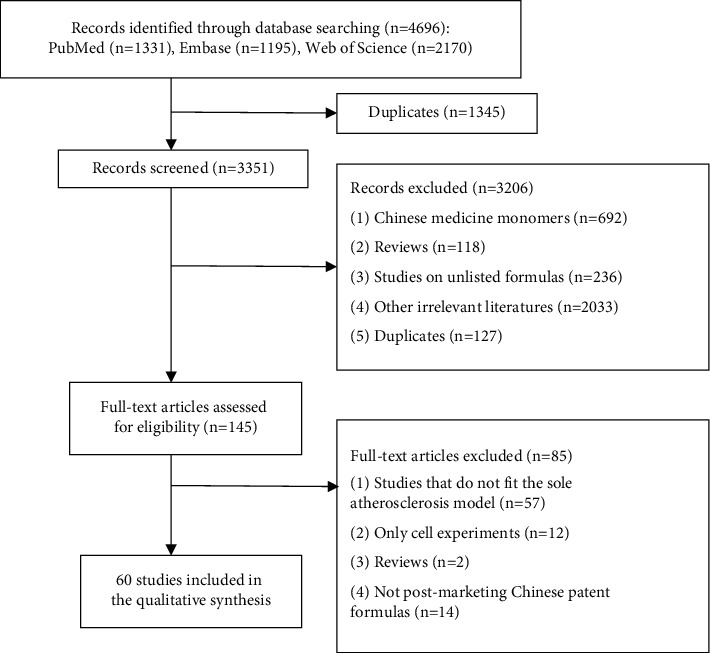
Flowchart of searching and screening studies.

A total of 4696 articles were retrieved from three literature databases. After removing 1345 duplicates, 3351 potentially relevant articles were assessed. Subsequently, 3206 articles were excluded after the evaluation of titles and abstracts. Of the 145 remaining articles, we further excluded 85 after screening the full text. Finally, 60 studies [19–40], [41–60], [61–78] were included in this analysis. A flowchart (Figure 1) shows the search and study selection process.

### 3.2. Study Characteristics

Herein, 60 studies [19–40], [41–60], [61–78], encompassing 23 postmarketing Chinese patent formulas, including Danhong injection, Zhixiong capsule, Longshengzhi capsule, Tongxinluo capsule, Shexiang Baoxin pill, Danlou tablet, Angong Niuhuang pill, Longxuetongluo capsule, Longhu Rendan, Naoxintong pill/capsule, Di'ao Xinxuekang capsule, Xuezhikang, Qishenyiqi pill, ginkgo biloba tablet, Xuefu Zhuyu granule, Shexiang Tongxin dropping pill, compound Chuanxiong capsule, Yindanxinnaotong soft capsule, Dahuang Zhechong pill, Fufang Danshen dropping pill, Suxiaojiuxin pill, Xuezhitong capsule, and Guanxinshutong capsule. All the studies utilized the validated AS models and were published from 2004 to 2020. The HFD feeding time varied from 0 to 34 weeks, and the duration of different drug treatments ranged from 12 days to 34 weeks. The characteristics of the included studies and the herbal drugs of the included formulas are shown in Table 1 and Supplementary Tables S1–S3.

### 3.3. ROB and Methodological Quality

According to the assessment of SYRCLE's ROB tool, the included animal intervention studies displayed the methodological bias of 10 entries shown in Supplementary Table S4. All the included studies had unclear baseline characteristics, allocation concealment, blinding method, and random outcome assessment. Among these, five low-risk studies [40, 42, 43, 66, 68] used the “random number table” in the assessment of sequence generation, seven high-risk studies [24, 33, 34, 49, 59, 75, 76] did not report the entry, and the remaining 48 studies had unclear detailed random methods. Although none of the studies mentioned random housing, the outcome measures in the 19 studies [20, 23, 26, 27, 29, 30, 34–36, 38, 41, 45, 46, 48, 50, 52, 59, 73, 75] were not influenced since the feeding conditions (such as temperature, lighting, and humidity) were described, and the bias was evaluated as low risk. The incomplete outcome data were detected in 27 high-risk articles [19–21, 23, 26, 32–36, 39, 45–47, 50–53, 55, 56, 60, 61, 68, 74–77] without any reason or appropriate method for describing the missing data, while 18 articles [22, 24, 25, 27–31, 38, 41, 49, 54, 57, 64, 70–72, 78] were unclear of the attrition bias. Although none of the studies provided any protocol, almost all had reported the expected outcome indicators, and most of them were evaluated as the low-risk bias. Moreover, other risks were not detected in the whole studies. The results showed that the methodology of the 60 studies included in this review was incomplete, and the quality was generally low.

### 3.4. Outcome Analysis

#### 3.4.1. Models of AS

A total of 15 primary AS models were utilized in these studies, including HFD ApoE^−/−^ mice, HFD LDLR^−/−^ mice, HFD New Zealand or Japanese rabbits, HFD SD rats, HFD SD or Wistar rats with vitamin D3 injection, HFD Wistar rats with vitamin D3 injection and balloon injury in aorta, SD rats with the carotid artery balloon injury, HFD New Zealand rabbits or Japanese rabbits with balloon injury in aorta, HFD ApoE^−/−^ mice with silastic collar implantation, Wistar rats with the silicone collar around the carotid artery, HFD Japanese rabbits or New Zealand rabbits with the silastic collar implantation around the carotid artery, C57BL/6 mice with the common carotid artery ligation, ApoE^−/−^ mice combined partial ligation of the left common carotid artery and left renal artery, HFD New Zealand rabbits with balloon injury and plaques triggering by Chinese Russell viper venom (RVV), and HFD New Zealand rabbits with balloon injury, transfected with adenovirus-containing p53 and plaques triggered by RVV.

Among these, mice and rabbits were common animal species that accounted for 70% of the included studies. The HFD ApoE^−/−^ mice model constituted 45% (27/60) of the studies, and rabbits made up 25% (15/60) of the studies. The models established by extra injection of adenovirus-containing p53 or triggered with RVV aimed to aggravate local inflammation and led to vulnerable plaques in rabbits [71, 72]. Despite the varied content of fat feeding, HFD was fed in most (93.3%, 56/60) of the animal models to promote the formation of AS. Furthermore, studies using only male animals constituted 81.7% (49/60) of the series.

#### 3.4.2. Amelioration of Plaque Area and Artery Structure

In summary, most of the included studies [19–24, 26, 28, 30–42, 44–52], [55, 58, 63–66, 68–72, 74–78] showed that the drugs, including 22 drugs of Danhong injection, Zhixiong capsule, Longshengzhi capsule, Tongxinluo capsule, Shexiang Baoxin pill, Danlou tablet, Angong Niuhuang pill, Longxuetongluo capsule, Longhu Rendan, Naoxintong pill/capsule, Di'ao Xinxuekang capsule, Xuezhikang, Qishenyiqi pill, ginkgo biloba tablet, Xuefu Zhuyu granule, Shexiang Tongxin dropping pill, compound Chuanxiong capsule, Yindanxinnaotong soft capsule, Fufang Danshen dropping pill, Suxiaojiuxin pill, Xuezhitong capsule, and Guanxinshutong capsule, significantly reduce the atherosclerotic plaque area (APA) in different AS models.

In addition to the reduction in APA, Zhixiong capsule altered the artery structure of attenuating intima thickening (IT), intimal area (IA), and IA/MA (intimal area/medial area) ratio [20, 78]; Danlou tablet reduced luminal occlusion (LO) and lipid content in the artery [24, 75, 77]; Naoxintong capsule, ginkgo biloba tablet, Tongxinluo capsule, and Fufang Danshen dropping pill also decreased LO [32, 37, 69, 70]; Angong Niuhuang pill attenuated IT and media thickness (MT) [35]; ginkgo biloba tablet, Danlou tablet, Tongxinluo capsule, Fufang Danshen dropping pill, and Xuefu Zhuyu granule reduced IT [37, 40, 46, 68]; Tongxinluo capsule decreased IA and the IA/MA ratio [49] and attenuated the external elastic membrane area (EEMA) and intima-media thickness (IMT) [71, 72].

#### 3.4.3. Modification of Blood Lipid Level

Among these studies, 20 formulas, including Danhong injection, Zhixiong capsule, Danlou tablet, Longxuetongluo capsule, Naoxintong, Qishenyiqi pill, Angong Niuhuang pill, ginkgo biloba tablet, Xuefu Zhuyu granule, Yindanxinnaotong capsule, Longhu Rendan, Di'ao Xinxuekang capsule, compound Chuanxiong capsule, Tongxinluo, Shexiang Tongxin dropping pill, Fufang Danshen dropping pill, Xuezhikang, Suxiaojiuxin pill, Xuezhitong capsule, and Guanxinshutong capsule, showed the potential lipid-lowing property.

Furthermore, Danhong injection significantly suppressed the serum total triglyceride (TG) and low-density lipoprotein cholesterol (LDL-C) levels, upregulated the serum high-density lipoprotein cholesterol (HDL-C) level of HFD ApoE^−/−^ mice [19], and downregulated the serum total cholesterol (TC), TG, and LDL-C levels of HFD New Zealand rabbits [63]. Zhixiong capsule administration showed a significant plasma lipid-lowering effect of the decreased TC level, LDL level, TC/HDL-C ratio, and log(TG/HDL-C) value and elevated HDL-C content [20, 78]. Danlou tablet also significantly reduced the TC, TG, and LDL-C levels in HFD ApoE^−/−^ mice [77] and HFD Wistar rats with vitamin D3 injection [42] and increased the HDL-C levels in HFD ApoE^−/−^ mice [24]. Longxuetongluo capsule decreased the serum TC, TG, and LDL-C levels and increased HDL-C levels in HFD male SD rats [26]. Naoxintong inhibited the serum LDL-C and TC in HFD New Zealand rabbits [57], reduced TC and TG in HFD LDLR^−/−^ mice [58], and promoted the HDL-C level in HFD ApoE^−/−^ mice [32]. Qishenyiqi pill decreased the level of blood LDL-C in HFD ApoE^−/−^ mice [34]. Angong Niuhuang pill significantly reduced the serum content of TC, LDL-C, and the ratio of LDL-C to HDL-C in HFD male SD rats with vitamin D3 injection [35]. Ginkgo biloba tablet treatment significantly reduced serum levels of TC, TG, and LDL-C in HFD Wistar male rats with vitamin D3 injection and balloon injury in the aorta [37]. Xuefu Zhuyu granule reduced the serum level of TC in HFD Wistar male rats with vitamin D3 injection [40]. Yindanxinnaotong capsule also decreased the levels of blood TC, TG, and LDL-C in HFD SD male rats with vitamin D3 injection [48]. Longhu Rendan, Di'ao Xinxuekang capsule, or compound Chuanxiong capsule treatment ameliorated AS by significantly reducing serum levels of TC, TG, and LDL-C in HFD ApoE^−/−^ mice [28, 30, 47]. Tongxinluo, Shexiang Tongxin dropping pill, Fufang Danshen dropping pill, Xuezhikang, Suxiaojiuxin pill, Guanxinshutong capsule, or Xuezhitong capsule treatment reduced serum levels of TC, TG, and LDL-C and increased HDL-C in different models [44, 52, 54, 66, 68, 72, 74, 76]. In addition, Danhong injection and compound Chuanxiong capsule decreased the atherosclerotic index (AI) [19, 47], which could be calculated as the ratio of non-HDL-C and HDL-C.

#### 3.4.4. Modification of Plaque Stability

To summarize, nine formulas, including Longshengzhi capsule, Tongxinluo capsule, Naoxintong, Danhong injection, Xuezhikang, compound Chuanxiong capsule, Suxiaojiuxin pill, Zhixiong capsule, and Guanxinshutong capsule, could stabilize the plaques in different models.

Longshengzhi capsule maintained the integrity of the arterial wall and enhanced plaque stability by decreasing necrotic core areas (NCAs); increasing collagen-positive areas (CPAs), smooth muscle cell (SMC) content, and fibrous cap areas (FCAs); and inhibiting cell apoptosis in the lesion areas [21]. Tongxinluo capsule treatment increased the stability of plaques by increasing the intraplaque contents of SMC, CPA, and fibrous cap thickness (FCT) and reducing that of macrophages and lipids [22, 41]. The vulnerability index (VI) was calculated as follows: (lipids staining% + macrophages (MOMA-2) staining%)/(SMCs staining% + collagen staining%). Tongxinluo capsule treatment also stabilized atherosclerotic plaques by significantly attenuating VI and macrophage apoptosis and enhancing Beclin-1-induced autophagy [31]. In the plaque rupture models, Tongxinluo capsule treatment prevented vulnerable plaques from rupture by downregulating VI, reducing macrophages and lipids, increasing CPA and SMC, lowering the levels of matrix metalloproteinase-1 (MMP-1), MMP-3, and MMP-12, and upregulating tissue inhibitor of metalloproteinase-1 (TIMP-1) protein in plaques [71, 72]. Furthermore, Tongxinluo capsule treatment downregulated MMP-9 [64] and MMP-2 in plaques [22]. Naoxintong treatment enhanced plaque stability by increasing CPA, FCT, FCA, and SMC content and reducing the MMP-2 level, macrophage accumulation, and calcification events in lesion areas [32, 39]. Danhong injection maintained the content of CPA in the arterial wall and reduced the expression of MMP-2 and MMP-9 [53]. Xuezhikang treatment also increased CPA and SMC and decreased NCA, MMP-8, and MMP-13, thereby stabilizing the atherosclerotic plaques and rupture by the suppression of macrophage endoplasmic reticulum (ER) stress-mediated apoptosis and the NF-*κ*B pathway [33]. Compound Chuanxiong capsule increased collagen proportion in plaques [47]. Suxiaojiuxin pill enhanced atherosclerotic plaque stability by increasing SMC, TIMP-1, and TIMP-2 proteins while decreasing MMP-2 and MMP-9 proteins, which might be associated with the mechanism of modulating the MMPs/TIMPs balance [50]. Both Zhixiong and Guanxinshutong capsules enhanced plaque stability by increasing the content of CPA [76, 78]. In addition, Zhixiong capsule decreased vascular mineralization [78] and Guanxinshutong reduced the accumulation of macrophages in aortic root sections [76].

#### 3.4.5. Amelioration of Lipid Metabolism and Lipid Accumulation

The disorder of hepatic lipid metabolism induced fatty liver and increased the risk of AS. Excessive accumulation of lipid in aortas and oxidative low-density lipoprotein (ox-LDL) taken in by macrophages could also lead to AS. A total of 14 formulas involving Longshengzhi capsule, Naoxintong, Shexiang Baoxin pill, Danlou tablet, Longhu Rendan, ginkgo biloba tablet, Di'ao Xinxuekang capsule, Xuezhitong capsule, Qishenyiqi pill, Danhong injection, Tongxinluo capsule, Shexiang Tongxin dropping pill, Zhixiong capsule, and Suxiaojiuxin pill described the related mechanisms.

Longshengzhi capsule ameliorated hepatic lipid metabolism by activating the sterol regulatory element binding protein (SREBP) 2 pathway and regulating the expression of SREBP1c protein, low-density lipoprotein receptor (LDLR), hydroxymethylglutaryl coenzyme A synthase (HMGCS), diacylglycerol acyltransferase-1 (DGAT1), adipose triglyceride lipase (ATGL), microsomal triglyceride transporter protein (MTTP), and apolipoprotein C II (APOC2) related to lipogenesis, cholesterol, and TG metabolism in the liver. Longshengzhi capsule also reduced AS lesions associated with the reduction of macrophage and foam cell accumulation by triggering the expression of ATP binding cassette transporter A1 (ABCA1) and ATP binding cassette transporter G1 (ABCG1) [21]. Naoxintong capsule reduced hepatic TG levels by the inhibition of TG synthesis and the activation of TG hydrolysis, including downregulation of DGAT1 while activating AMPK*α*, ATGL, and comparative gene identification-58 (CGI-58) expression in the liver [32]; Naoxintong treatment also significantly reduced foam cell accumulation in atherosclerotic plaques [29, 32]. Shexiang Baoxin pill treatment inhibited lipid accumulation by elevating the levels of liver X receptor *α* (LXR*α*), ABCA1, and ABCG1 and reducing the content of scavenger receptor class A (SR-A) and lectin-like oxidized low-density lipoprotein receptor-1 (LOX-1) in the arterial wall [23]. The mechanism of Danlou tablet in accelerating cholesterol efflux was to activate the peroxisome proliferator-activated receptor *α* (PPAR*α*)/ABCA1 signaling pathway by upregulating the expression of PPAR*α*, PGC-1*α*, and ABCA1 [24]. Longhu Rendan ameliorated AS via downregulating the protein expression of LOX-1 in the aortic root, subsequently attenuating AS and lipid deposition [28]. Ginkgo biloba tablet decreased the content of SR-A in the arterial wall [37]. Di'ao Xinxuekang capsule treatment demonstrated lipid-lowering and antiatherosclerotic mechanisms via the downregulation of proprotein convertase subtilisin/kexin type 9 (PCSK9) and upregulation of the LDLR signaling pathway in the liver tissue [30]. It also facilitated reverse cholesterol transport (RCT) via enhanced cholesterol efflux through ABCA1 and ABCG1 in aortas, the upregulation of HDL synthesis modulated by the PPAR*γ*-LXR*α*-ABCA1 pathway, the modification of HDL maturation by increasing serum lecithin-cholesterol acyltransferase (LCAT) activity, and the promotion of scavenger receptor class B type 1 (SR-B1)-mediated HDL-cholesteryl ester uptake [33]. Xuezhitong capsule improved blood lipid dysfunction via the activation of RCT and the accompanying increase in the HDL levels, as characterized by improved ABCA1, SR-B1, LCAT, apolipoprotein A I (ApoA1), and apolipoprotein B (ApoB) [74]. Qishenyiqi pill treatment removed blood cholesterol by promoting the LDLR-LXR-*α*-ABCG5 pathway in the liver, and it also blocked phagocytosis of ox-LDL by macrophages by inhibiting the expression of CD36 in the aorta [34]. Danhong injection treatment reduced the AS by inhibiting HMG-CoA reductase (HMGCR), activating the LDLR in the liver, and reducing macrophage accumulation while increasing ABCA1 expression in the aortic root in various AS models [55]. Tongxinluo capsule administration promoted the antiatherosclerotic effects by increasing the level of PPAR*γ* in aortas [64] and lowering the expression of macrophages and LOX-1 in vascular walls [65, 67, 72]. Zhixiong capsule blocked the proliferation of macrophages and monocytes, thereby reducing the formation of foam cells by upregulating p53 expression and decreasing MAPK14 expression [78]. Moreover, all these formulas, including Danlou tablet, Shexiang Tongxin dropping pill, Suxiaojiuxin pill, Tongxinluo capsule, and Xuezhitong capsule treatment, led to a significant reduction in the serum level of ox-LDL [42, 44, 61, 72, 74].

#### 3.4.6. Anti-inflammation and Antioxidant Stress

The development and progression of AS were associated with oxidative stress and chronic inflammation. In summary, a total of 18 formulas, including Zhixiong capsule, Longshengzhi capsule, Tongxinluo capsule, Shexiang Baoxin pill, Danlou tablet, Angong Niuhuang pill, Longxuetongluo capsule, Xuezhikang, Qishenyiqi pill, ginkgo biloba tablet, Naoxintong capsule, Shexiang Tongxin dropping pill, compound Chuanxiong capsule, Yindanxinnaotong capsule, Danhong injection, Fufang Danshen dropping pill, Suxiaojiuxin pill, and Guanxinshutong capsule, have potential anti-inflammatory properties. Nine formulas, including Tongxinluo capsule, Shexiang Baoxin pill, Angong Niuhuang pill, Xuezhikang, Shexiang Tongxin dropping pill, Yindanxinnaotong capsule, Dahuang Zhechong pill, Suxiaojiuxin pill, and Guanxinshutong capsule, were related to antioxidant stress functions.

Zhixiong capsule intervened AS progression by blocking the proinflammatory process and increasing the plasma level of interleukin-4 (IL-4) and IL-13 [20, 78]. Longshengzhi capsule reduced AS lesions related to potent anti-inflammatory effects, including decreasing the expression of serum tumor necrosis factor-*α* (TNF-*α*), the number of Kupffer cells, and the levels of C-C chemokine receptors-2 (CCR2), IL-6, monocyte chemoattractant protein-1 (MCP-1), and TNF-*α* in liver sections [21]. Tongxinluo capsule inhibited inflammation by reducing the expressions of IL-6, MMP-2, IL-1*β*, intercellular adhesion molecule-1 (ICAM-1), MCP-1, vascular cell adhesion molecule-1 (VCAM-1), and TNF-*α* in the arterial wall [22, 27, 49, 51, 52, 62, 71, 72]. Moreover, Tongxinluo also decreased the serum proinflammatory levels of IL-8, IL-18, high-sensitivity C-reactive protein (hs-CRP), MCP-1, MMP-1, ICAM-1, and VCAM-1 [51, 52, 62, 71, 72]. It may also reduce inflammation by inhibiting NF-*κ*B expression in arteries [27, 45, 72]. Shexiang Baoxin pill regulated the serum levels of proinflammatory and anti-inflammatory cytokines, including MCP-1, interferon-*γ* (IFN-*γ*), IL-17A, IL-10, and transformed growth factor-*β*1 (TGF-*β*1), but decreased the levels of proinflammatory factors, including VCAM-1, ICAM-1, IL-6, and IL-2, in the vascular wall; moreover, it suppressed the activities of inflammation-related pathways by elevating the level of Mfn2 and reducing the phosphorylation of NF-*κ*B, JNK, and p38 in the aorta [23]. Danlou tablet treatment attenuated AS by downregulating the NF-*κ*B signaling pathway and decreasing the levels of IL-1*β*, MCP-1, IL-18, and IL-33 in plaques [24, 77] and suppressing the serum levels of IL-6, TNF-*α*, MCP-1, IL-8, MMP-1, MMP-2, ICAM-1, TNF-*α*, IL-1*β*, lipoprotein-associated phospholipase A2 (LP-PLA2), and secretory phospholipase A2 (sPLA2) in the aorta [42, 75]. Angong Niuhuang pill prevented AS associated with the anti-inflammatory effects and the immunoregulatory functions via multiple targets: the ratio of splenic T helper 17 cells (Th17) to regulatory T cell (Treg), cytokines (IL-6, TGF-*β*1, and IL-17), chemokines (MCP-1, MCP-2, MCP-3, CCR2, and CXCR3), cell adhesion molecules (ICAM-1 and VCAM-1) in the aorta [25], and serum CRP [35]. The underlying mechanism of the Longxuetongluo capsule for AS may be attributed to its anti-inflammatory effects of reducing serum levels of VCAM-1, ICAM-1, and MCP-1 and decreasing NF-*κ*B expression in the aorta [26]. Xuezhikang treatment was also associated with the NF-*κ*B proinflammatory pathway in ApoE^−/−^ mice combined with artery ligations [33]. Qishenyiqi pill inhibited AS by promoting Treg immigration into atherosclerotic plaques and inhibiting the secretion of IL-17 via Th17 in the spleen and plaques [34]. Ginkgo biloba tablet also promoted the anti-inflammatory effects in reducing serum CRP, ICAM-1, and VCAM-1 levels [37]. Naoxintong capsule inhibited the expression of proinflammatory molecules MMP-2 and TNF-*α* in the aorta [39]. Shexiang Tongxin dropping pill treatment significantly decreased the serum levels of the proinflammatory cytokines IL-2, IL-6, TNF-*α*, and INF-*γ* [43, 44]. Compound Chuanxiong capsule prevented AS by regulating the PI3K/Akt/NF-*κ*B signaling pathway and inhibiting the expression of IL-6 and TNF-*α* [47]. Yindanxinnaotong capsule relieved AS lesions by repressing the inflammation activity of inhibiting the NF-*κ*B signaling pathway and serum proinflammatory cytokines IL-1*β*, CRP, and TNF-*α* [48]. Danhong injection administration reduced the proinflammatory cytokines, MCP-1 [53] and TNF-*α* [55]. Fufang Danshen dropping pill reduced VCAM-1 in the aorta [69], and Suxiaojiuxin pill reduced NF-*κ*B protein expression in the aorta [61]. Guanxinshutong capsule also regulated AS progression by inhibiting TNF-*α*, IL-6, and NF-*κ*B expression [76].

Tongxinluo inhibited oxidative stress injury by downregulating serum malondialdehyde (MDA), while upregulating the levels of nuclear factor erythroid-2-related factor 2 (Nrf2) and NADPH quinone oxidoreductase-1 (NQO1) in the arterial wall and increasing the serum levels of superoxide dismutase (SOD) and total antioxidant capacity (T-AOC) [27]. Shexiang Baoxin pill enhanced the antioxidative abilities by increasing SOD, catalase (CAT), and glutathione (GSH) in the circulation of ApoE^−/−^ mice and improved the oxidative injury by reducing serum MDA, hydrogen peroxide (H_2_O_2_), and myeloperoxidase (MPO) content [23]. Angong Niuhuang pill also exerted the antioxidant effect of decreasing the serum MDA level [35]. Xuezhikang regulated the blood levels of MDA, SOD, CRP, and T-AOC [56, 59, 66]. Shexiang Tongxin dropping pill increased the serum levels of GSH and SOD and decreased MDA, which also reduced reactive oxygen species (ROS) generation in the aortic root lesions [43, 44]. Yindanxinnaotong capsule enhanced the antioxidative effect by regulating SOD, MDA, GSH, and glutathione peroxidase (GSH-PX) [48]. Guanxinshutong capsule improved serum SOD, MDA, and GSH and upregulated the expression of heme oxygenase-1 (HO-1) and Nrf2 in the aortic sinus [76]. Both Dahuang Zhechong and Suxiaojiuxin pills attenuated AS via the regulation of SOD and MDA levels in the serum [60, 61] and inhibited MPO in the arterial wall [60].

#### 3.4.7. Other Effects and Mechanisms

Other mechanisms were mainly related to improved endothelial dysfunction, the inhibition of angiogenic factors, the regulation of VSMCs proliferation, and apoptosis.

Tongxinluo administration alleviated early AS and inhibited adventitial vasa vasorum (VV) angiogenesis by regulating the angiogenic expression of vascular endothelial growth factor-A (VEGF-A), its receptor VEGF-R2, angiopoietin-1 (ANGPT-1), and CD34 in the artery [27, 41, 46]. Tongxinluo also inhibited neointimal hyperplasia via the overexpression of miR-155 [49]. Moreover, Tongxinluo attenuated the chronic vasoconstriction in the regulation of neuronal nitric oxide synthase (nNOS) by stimulating the ERK1/2 signaling pathway [70]; it also improved endothelial functions by reducing serum ET-1 and upregulated nitric oxide (NO) [51]. In addition, Tongxinluo also inhibited monocyte adhesion via reduced plasminogen activator inhibitor type-1 (PAI-1) [62]. Xuezhikang inhibited AS lesions probably by elevating endothelial nitric oxide synthase (eNOS)/NO production in the aortic wall and improving hemorheology [56]; it also reduced blood hypercoagulation and inhibited the tissue factor expression [59]. Dahuang Zhechong pill inhibited AS by exerting a protective effect on vascular endothelium, inhibiting VSMCs proliferation, and promoting VSMC apoptosis [60]. Both Danlou tablet and Xuefu Zhuyu granule decreased the serum level of platelet-derived growth factor (PDGF) and inhibited ERK1/2 signaling pathway activation and VSMCs proliferation [40]. Shexiang Baoxin pill reversed the dedifferentiation of VSMCs from the synthetic phenotype to the contractile phenotype [73]. Danhong injection treatment ameliorated HFD-induced AS and insulin resistance by activating the PI3K/AKT insulin pathway induced by insulin receptor substance-1 (IRS-1) [19]. The Danhong injection treatment also protected the endothelial function by inhibiting the expression of inducible nitric oxide synthase (iNOS) and COX-2 in the aortic wall [63]. Yindanxinnaotong inhibited AS on the vascular endothelial function markers, including thromboxane B2 (TXB2) and NO [48]. Shexiang Tongxin dropping pill regulated the expression of miR-21, miR-126, miR-155, miR-132, and miR-20 in the aorta, which might attenuate AS [43, 44]. Naoxintong capsule also showed potent atheroprotective power in reducing iNOS expression in AS lesions and inhibiting dendritic cell (DC) maturation [57, 58].

## 4. Discussion

In this review, we have summarized 60 studies, including 23 Chinese patent formulas in treating AS. The animal models, effects, and mechanisms were listed via a critical systematic review. Opposite to Western medicine, traditional Chinese herbs are aimed at multiple targets for various pharmaceutical ingredients. TCM also has the potential to increase the therapeutic efficacy of AS.

### 4.1. Choice of AS Models

Herein, we presented well-established animal models related to AS. The HFD ApoE−/− mice pattern might be a long-desired AS model owing to its high plasma TC level, foam cell-rich depositions in the proximal aortas, and easy breeding and handling procedures [79, 80]. Rabbits are also commonly used in AS models; both ApoE^−/−^ mice and rabbits are suitable models for the studies of plaque stability and lipid accumulation in vascular walls. Despite the sensitivity to dietary cholesterol induction, the locations of AS lesions in rabbits are different from those of humans [81], and prolonged cholesterol feeding in rabbits may result in hepatic toxicity [82]. The major limitation of ApoE^−/−^ mice is the rare occurrence of thrombosis and plaque rupture, which is common in humans [16]. Owing to the studies of vulnerable plaques, the rabbit model is successfully established through the overexpression of p53 in local plaques and triggered by RVV [83]. Rats and C57BL/6 mice are used in the included studies; these animals are naturally resistant to atherogenesis and lack plasma cholesteryl ester transfer protein (CETP) activity [79, 84]. In addition, the rat and mouse models established by HFD, vitamin D3 injection, balloon injury, and artery ligation may be valuable methods to promote AS; however, the fibrous plaques in the vascular wall lack lipid depositions and are different from those of ApoE^−/−^ mice and rabbits [79]. Typically, there is no perfect model for any experimental condition, and an appropriate animal model should be considered based on the sample size, docility, feeding and housing, specific genetic profile, aims of the pathological aspects, costs, and analogy with humans [85].

### 4.2. Methodological Quality of the Results

We also assessed the methodological quality of the included studies and evaluated all of them as generally low. Although many entries were judged as unclear risk of bias, the reporting of essential details still needs to be emphasized. Sequence generation, baseline characteristics, and random outcome assessment are necessary to be reported in an animal experiment in order to make the results adequately comparable. Regarding random housing conditions, the pharmacological agents could be influenced because of inconsistent temperature, lighting, humidity, and nonrandomized shelves or different rooms for animals [18]. For the judgment of attrition bias, the incomplete outcome data should be noticed and addressed adequately. SYRCLE's ROB is an adapted tool based on the Cochrane ROB tool that facilitates critical appraisal in a systematic review and improves the reporting quality of animal experiments. However, during the actual animal experiment, random methods should be considered with respect to animal weight, modeling parameters, and high-fat intake that would impact grouping. Although it is an effective method to improve internal validity and avoid subjectivity in interpreting the results, the blinding of caregivers, investigators, and outcome assessors in animal studies is often hard to achieve. Considering the practical difficulties in some items, we propose that SYRCLE's ROB could be further updated to better evaluate the animal experiments.

### 4.3. Effects and Mechanisms in All Formulas

Based on the outcome analysis, we summarized the effects and mechanisms involved in plaque formation and stability, the changes in the lipid level, lipid metabolism and accumulation, anti-inflammation, and antioxidative stress *in vivo*. Interestingly, most of the included drugs in different models reduce the areas of plaques, which are directly related to AS procession. Regarding the stability of plaques, the relevant factors are the areas of plaque necrotic core and fibrous cap, the content of collagen and SMCs, macrophage accumulation and apoptosis, and the synthesis and balance of MMPs/TIMPs in lesion areas. In addition, hyperlipidemia is the independent risk in AS, and over three-quarters of the drugs have reported the lipid-lowing property related to the downregulation of TC, TG, and LDL-C and upregulation of HDL-C in the serum level. The in-depth studies further found the mechanisms in the modification of hepatic lipid disorders and promoted lipid efflux from macrophages, which reduce the lipid deposition and indirectly inhibit AS development. Excessive phagocytosis of ox-LDL into macrophages depends on the scavenger receptors, such as SR-A, CD36, and LOX-1, and ultimately leads to foam cell formation and AS procession. In the current review, some formulas have also been identified for the property of reducing the scavenger receptors. In addition, chronic inflammation and oxidative stress are the major contributors throughout the whole AS progression [86, 87]; some formulas have been verified to suppress the proinflammatory and pro-oxidative stress while activating the anti-inflammation and antioxidative stress, including the NF-*κ*B signaling pathway and the related cytokines, chemokines, cell adhesion molecules, and oxidant enzymes. Moreover, some studies have listed other atheroprotective mechanisms in protecting endothelial injury and inhibiting VSMCs proliferation and migration.

### 4.4. Multiple Components and Targets in Chinese Patent Formulas

Statins are the first choice for AS that can reduce plasma cholesterol levels due to their effects on the synthesis, reuptake, and intestinal absorption of cholesterol [88]. Different from statins, Chinese patent formulas contain many active components with multiple targets and functions in AS. In this review, 16 studies of Tongxinluo capsules elucidated the effects and mechanisms of ameliorating APA, recovering the serum lipid level, inhibiting macrophage accumulation, stabilizing atherosclerotic plaques, reducing inflammation, and antioxidant stress, and protecting endothelial dysfunction in ApoE^−/−^ mice and rabbit models. Especially in the vulnerable plaque rabbit model, Tongxinluo capsule treatment exerts protective effects in plaque rupture, and the mechanisms in the amelioration of MMPs and TIMPs were explored [31]. The bioactive ingredients from Tongxinluo also supported the evidence in the prevention and treatment of AS. According to the major extracts, ginsenoside Rb1 has anti-AS effects in reducing inflammation and oxidative stress responses, stabilizing plaques and attenuating the plaque formation. Additionally, it prevents endothelial dysfunction by upregulating the eNOS expression and improves the balance between apoptosis and autophagy; ginsenoside Rg1 has also been reported to inhibit AS by activating the AMPK/mTOR signaling pathway in the macrophages [89, 90]. Paeoniflorin is one of the aqueous extracts of Tongxinluo that could ameliorate AS by inhibiting the inflammation of the TLR4/MyD88/NF-*κ*B pathway [91]. The combination of bioactive ingredients in a formula has a synergistic role in treating AS and is beneficial to the balance of the whole body.

Unlike other formulas, Xuezhikang extract from red yeast Chinese rice containing lovastatin, unsaturated fatty acids, essential amino acids, and ergosterol [59] naturally. In addition to the lipid-lowering and anti-inflammatory effects of lovastatin, Xuezhikang has also been investigated with respect to stabilizing atherosclerotic plaques and improving endothelial dysfunction, which might be related to other useful substances [33, 56]. In our study, other formulas like Danhong injection, Danlou tablet, and Naoxintong capsule were also significant. Besides the mechanisms in reducing APA and blood lipids, inhibiting plaque inflammation, and regulating the level of oxidative stress, Danhong injection can maintain the collagen content in the arterial wall and reduce the expression of MCP-1, MMP-2, and MMP-9 mRNAs in the aortic wall [53]; ethanol extracts of Danlou tablet plays a key role in anti-inflammation and preventing lipid deposition in macrophages of AS via suppressing the NF-*κ*B signaling pathway and triggering the PPAR*α*/ABCA1 signaling pathway [24]; Naoxintong has shown its ability in inhibiting dendritic cells maturation, improving endothelial function, and enhancing plaque stability [29, 32, 39, 57, 58]. In summary, multiple components with multiple targets in Chinese patent formulas are beneficial in AS and may surpass single Western therapies.

### 4.5. Limitations

Nevertheless, the present study has several limitations. Firstly, we used the qualitative synthesis rather than the quantitative method due to the varied models and inconsistent interventions, and hence, we could not draw specific conclusions from the meta-analysis. Owing to the stringent inclusion and exclusion criteria in the systematic review, relevant cell experiments were excluded, necessitating further investigation of the mechanisms *in vitro*. In addition, to elucidate the effects and mechanisms of action, HPLC/MS analysis for compound identification should be employed.

## 5. Conclusions

In summary, our analysis revealed the atheroprotective effects and mechanisms of 23 postmarketing Chinese patent formulas *in vivo*. In this systematic review, we first summarized the roles of the amelioration of plaques, the improvement of plaque stability, the modification of lipid level and metabolisms, and the anti-inflammation and antioxidant stress in alleviating AS lesions. Also, the methodological quality was found to be low in the included literature. Thus, additional high-quality evidence is essential through a broad perspective of postmarketing Chinese patent formulas in treating AS.

## Figures and Tables

**Table 1:** Details of postmarketing Chinese patent formulas in the process of atherosclerosis *in vivo*.

Author, year	Postmarketing Chinese patent formulas	Experimental models	HFD feeding time	Drug intervention time	Outcome measures	Effects and mechanisms
Zhou et al. 2019 [19]	Danhong injection	HFD male apoE−/− mice	12 weeks	6 weeks	TG↓ HDL-C↑ LDL-C↓; APA↓ AI↓; NEFA↓ FBG↓ FINS↓ IR↓; GLUT-4↑ p-IRS-1↑ p-AKT↑	Attenuating AS and macrophage lipid accumulation by promoting the activation of PI3K/AKT insulin signaling pathway.
Zhai et al. 2019 [20]	Zhixiong capsule	HFD male Japanese rabbits with the silastic collar implantation around the right carotid artery	12 days	12 days	TC↓ HDL-C↑ TC/HDL-C ratio↓ log(TG/HDL-C)↓; IL-4↑; APA↓ IA↓ IA/MA↓	Preventing atherosclerotic plaque formation and intimal thickening.
Ma et al. 2019 [21]	Longshengzhi capsule	HFD female apoE−/− mice	18 weeks	10 weeks	Artery sections: APA↓; MOMA-2↓; NCA↓ CPA↑ FCA↑ SMC↑; TUNEL↓; ABCA1↑ ABCG1↑. Liver sections: lipid droplets↓ liver TG↓; FA oxidation↑ FA synthesis↓ SREBP1↓; SREBP2↓ LDLR↑ HMGCS↓; DGAT1↓ ATGL↑ MTTP↓ APOC2↑; CCR2↓ IL-6↓ MCP-1↓ TNF-*α*↓; CD68↓ MOMA-2↓. Serum TNF-*α*↓	Reducing AS by reducing macrophage/foam cell accumulation, maintaining the integrity of arterial wall, ameliorating hepatic lipid metabolism, and inhibiting inflammation.
Ma et al. 2019 [22]	Tongxinluo capsule	HFD male apoE−/− mice	16 weeks	16 weeks	APA↓; CPA↑ SMC↑ staining of lipids and macrophages↓; IL-6↓, MMP-2↓, and TNF-*α*↓	Inhibiting AS development and stabilizing plaque.
Lu et al. 2019 [23]	Shexiang Baoxin pill	HFD apoE−/− mice	20 weeks	20 weeks	APA↓; SOD↑ CAT↑ GSH↑ MDA↓ H_2_O_2_↓ MPO↓; MCP-1↓ IFN-*γ*↓ IL-17A↓ IL-10↑ TGF-*β*1↑; VCAM-1↓ ICAM-1↓ IL-6↓ IL-2↓; macrophages↓ ABCA1↑ ABCG1↑; p38↓ JNK↓ Mfn2↑ NF-*κ*B↓ SR-A↓ LOX-1↓ LXR*α*↑	Exerting antiatherosclerotic effects via improving inflammation response and inhibiting lipid accumulation.
Hao et al. 2019 [24]	Danlou tablet	HFD male apoE−/− mice	20 weeks	12 weeks	APA↓; LO↓; lipid content in artery↓ HDL-C↑ ox-LDL↓; IL-1*β*↓, IL-10↓, MCP-1↓, IL-18 ↓, IL-33↓; PPAR*α*↑, PGC-1*α*↑, ABCA1↑, P-IKK*α*/*β*↓, P-I*κ*B*α*↓, and P-NF-*κ*Bp65↓	Preventing AS via suppressing NF-*κ*B signaling and triggering the PPAR*α*/ABCA1 signaling pathway.
Chai et al. 2019 [25]	Angong Niuhuang pill	HFD male apoE−/− mice	8 weeks	8 weeks	Aorta: MCP-1↓ MCP-2↓ MCP-3↓ CCR2↓ CXCR3↓; ICAM-1↓ VCAM-1↓; IL-6↓ TGF-*β*1↑ IL-17↓; Treg cell↑; Th17/Treg cell↓. Spleen: IL-6↓ TGF-*β*1↑	Ameliorating the development of early AS by reducing splenic and vascular inflammation.
Zhou et al. 2018 [26]	Longxuetongluo capsule	HFD male SD rats	4 weeks	4 weeks	TC↓ HDL-C↑ LDL-C↓ TG↓; serum ALT↓ AST↓ serum MCP-1↓ ICAM-1↓ VCAM-1↓. Histological sections of liver and aorta↓; aortic histological sections: NF-*κ*B↓	Preventing AS and fatty liver by controlling lipid metabolism and anti-inflammation activity.
Yin et al. 2018 [27]	Tongxinluo capsule	HFD male and female New Zealand rabbits with the silicone tube encapsulation of left carotid artery	4 weeks	4 weeks	TC↓ TG↓ LDL-C↓; serum MDA↓, SOD↑, and T-AOC↑; VEGF-A↓ VEGF-R2↓; nuclear NF-*κ*B↓ TNF-*α*↓ IL-6↓; nuclear Nrf2↑ NQO1↑	Reducing carotid adventitial VV angiogenesis and alleviating early AS lesions by inhibiting carotid inflammation and oxidative stress injury.
Yan et al. 2018 [28]	Longhu Rendan	HFD male apoE−/− mice	10 weeks	10 weeks	TC↓ LDL-C↓ TG↓; APA↓; LOX-1↓	Ameliorating AS via reducing serum lipid and LOX-1 expression.
Wang et al. 2018 [29]	Naoxintong pill	HFD male apoE−/− mice	8 weeks	8 weeks	Plaque foam cell content↓	Reducing foam cell accumulation in atherosclerotic plaques.
Qu et al. 2018 [30]	Di'ao Xinxuekang capsule	HFD male apoE−/− mice	18 weeks	18 weeks	TC↓ HDL-C↑ LDL-C↓ TG↓; APA↓. Liver sections: lipid accumulation↓; PCSK9↓; liver LDLR↑ serum PCSK9↓	Alleviating lipid disorder and ameliorating AS with downregulation of the PCSK9.
Chen et al. 2018 [31]	Tongxinluo capsule	HFD male apoE−/− mice with silastic collar implantation	8 weeks	8 weeks	TUNEL↓ Lc3b dots↑; APA↓; vulnerable index↓ macrophage apoptosis↓	Improving autophagy via Beclin-1.
Yang et al. 2017 [32]	Naoxintong capsule	HFD male apoE−/− mice	18 weeks	8 weeks	APA↓ LO↓; HDL-C↑; CPA↑ SMC↑ MPO↑ CD68↓ calcification events↓ fibrous cap thickness↑. Liver sections: SREBP1↑ and SREBP2↑; ATGL↑ and LDLR↑; liver TG↓; DGAT1↓ CGI-58↑; ATGL↑ pi-AMPKa↑	Inhibiting AS development, stabilizing plaque, and reducing hepatic triglyceride levels.
Shen et al. 2017 [33]	Xuezhikang	Female apoE−/− mice combined partial ligation of the left common carotid artery and left renal artery	0	8 weeks	APA↓; CD68↓ *α*-SMA↑ CPA↑ vulnerable phenotype↓; p-PERK↓ p-IRE1*α*↓ p-eIF2*α*↓ and BiP↓ CHOP↓ DHE staining↓; NCA↓ TUNEL↓ caspase-3↓; TNF-*α*↓ MMP-8↓ MMP-13↓	Suppressing vulnerable plaque progression and rupture by mitigating lesional endoplasmic reticulum stress and inhibiting apoptosis and the NF-*κ*B proinflammatory pathway.
Peng et al. 2017 [34]	Qishenyiqi pill	HFD male apoE−/− mice	8 weeks	8 weeks	APA↓ LDL-C↓ liver weight/body weight↓. Liver sections: LXR*α*↑ ABCG5↑. Aorta sections: CD36↓ Foxp3↑ IL-17A↓. Spleen sections: Foxp3↓ IL-17A↓ Smad2/3↓ IL-6↓ ROR*γ*↓	Promoting regulatory T cells in atherosclerotic lesion, inhibiting T helper 17 cells in plaque and spleen, and accelerating liver cholesterol excretion.
Fu et al. 2017 [35]	Angong Niuhuang pill	HFD male SD rats with vitamin D3 injection	17 weeks	9 weeks	APA↓ IT↓ MT↓, the maximum platelet aggregation rates↓; serum TC↓ LDL-C↓ TC/HDL-C↓ LDL-C/HDL-C↓; MDA↓ hs-CRP↓ LDH↓ cTnI↓; myocardial fibers↓; Bax protein↓ Bcl-2↑	Reducing AS due to its antiplatelet aggregation, lipid regulatory, antioxidant, anti-inflammatory, and antiapoptotic properties.
Dong et al. 2017 [36]	Di'ao Xinxuekang capsule	HFD male apoE−/− mice	8 weeks	8 weeks	TC↓ LDL-C↓; APA↓. Aorta sections: ABCA1↑ and ABCG1↑. Liver and intestines: ABCA1↑ ApoA-I↑ PPAR*γ*↑ LXR*α*↑. Liver sections: SR-B1↑ preB1-HDL↓ HDL3↓ HDL2↑. Serum LCAT↑	Regulating RCT by improving HDL synthesis, maturation, and catabolism.
Zhu et al. 2016 [37]	Ginkgo biloba tablet	HFD male Wistar rats with vitamin D3 injection and balloon injury in aorta.	60 days	60 days	Blood glucose and calcium↓; TC↓ TG↓ LDL-C↓; LO↓ IT↓; SR-A↓; CRP↓ ICAM-1↓ VCAM-1↓	Alleviating AS lesions by inhibiting inflammation and controlling lipid.
Zheng et al. 2016 [38]	Longxuetongluo capsule	HFD male apoE−/− mice	6 weeks	6 weeks	APA↓	Reducing plaques.
Yang et al. 2016 [39]	Naoxintong capsule	HFD male apoE−/− mice	18 weeks	8 weeks	APA↓ CPA↑ SMC↑ calcification events↓ fibrous cap thickness↑ MOMA-2 protein↓ MMP-2↓ and TNF-*α*↓ SM22*α*↑	Reducing advanced AS and enhancing the plaque stability.
Miao et al. 2016 [40]	Danlou tablet/Xuefu Zhuyu granule	HFD male Wistar rats with vitamin D3 injection	4 weeks	8 weeks	Danlou group: IT↓ TC↓ TG↓ LDL-C↓ PDGF↓ ERK1/2↓ pERK1/2↓. Xuefu Zhuyu group: IT↓ TC↓ PDGF↓ ERK1/2↓ pERK1/2↓	Reducing serum lipids, increasing PDGF, and inhibiting ERK signal pathway activation and VSMC proliferation.
Ma et al. 2016 [41]	Tongxinluo capsule	HFD male apoE−/− mice	5 weeks	5 weeks	VEGF-A↓ ANGPT-1↑; microvessels sprouting↓ VV number in plaques↓; APA↓; CPA↑ SMC↑ MOMA-2↓ FCT↑	Inhibiting early AS through regulating angiogenic factor expression and inhibiting VV proliferation in atherosclerotic plaque.
Chen et al. 2016 [42]	Danlou tablet	HFD male Wistar rats with vitamin D3 injection	4 weeks	8 weeks	TC↓ TG↓ LDL-C↓; APA↓; IL-6↓ TNF-*α*↓ MCP-1↓ ox-LDL↓; LP-PLA2↓ sPLA2↓	Inhibiting AS related to the reduction of blood lipid and inflammation.
Xiong et al. 2015 [43]	Shexiang Tongxin dropping pill	HFD male apoE−/− mice	8 weeks	8 weeks	IL-2↓ IL-6↓ TNF-*α*↓ INF-*γ*↓ ox-LDL↓ MDA↓; GSH↑ SOD↑; ROS↓; miR-21↓ miR-126↓ miR-155↓ miR-20↑	Inhibiting AS via reducing inflammation and regulating miR-21, miR-126, miR-155, and miR-20.
Xiong et al. 2015 [44]	Shexiang Tongxin dropping pill	HFD male apoE−/− mice	8 weeks	8 weeks	APA↓; TC↓ TG↓ LDL↓ ox-LDL↓ HDL↑; IL-2↓ IL-6↓ TNF-*α*↓ INF-*γ*↓ ox-LDL↓ MDA↓; GSH↑ SOD↑ ROS↓; miR-21↓ miR-126↓ miR-155↓ miR-132↓ miR-20↑	Inhibiting AS via reducing inflammation and regulating miR-21, miR-126, miR-155, miR-132, and miR-20.
Wu et al. 2015 [45]	Tongxinluo capsule	HFD male apoE−/− mice	12 weeks	12 weeks	APA↓; p22↓ p47↓ HO-1↓; NF-*κ*B↓; TC↓ TG↓ LDL↓	Decreasing atherosclerotic plaque formation and inhibiting oxidative stress and inflammation.
Lang et al. 2015 [46]	Tongxinluo capsule	HFD New Zealand rabbits with the silastic collar implantation around the right carotid artery	4 weeks	4 weeks	IT↓; CD34↓; microvascular blood flow volume↓; VAGF↓ VEGFR-2↓	Inhibiting VV proliferation.
Kang et al. 2015 [47]	Compound Chuanxiong capsule	HFD male apoE−/− mice	13 weeks	7 weeks	TC↓ TG↓ LDL-C↓; AI↓ APA↓; CPA↑; PI3K↓ Akt↓ NF-*κ*B↓ IL-6↓ TNF-*α*↓	Preventing AS and inhibiting the expression of IL-6 and TNF-*α* by regulating the PI3K/Akt/NF-*κ*B signaling pathway.
Cheng et al. 2015 [48]	Yindanxinnaotong soft capsule	HFD male SD rats with vitamin D3 injection	9 weeks	12 weeks	APA↓ TC↓ TG↓ LDL-C↓; MDA↓ SOD↑ GSH↑ GSH-px↑; NF-*κ*B↓ IkB↑; IL-1*β*↓ CRP↓ TNF-*α*↓; NO↑ TXB2↓	Relieving AS through regulating lipids, reducing lipid particle deposition in the endothelial layer of artery, enhancing antioxidant power, and repressing inflammation activity by inhibiting the NF-*κ*B signal pathway.
Zhang et al. 2014 [49]	Tongxinluo capsule	Male C57BL/6 mice with the left common carotid artery ligation	0	21 days	IA/MA ratio↓ IA↓; TNF-*α*↓ IL-1*β*↓; miR-155↓	Inhibiting the vascular inflammatory response and neointimal hyperplasia.
Zhang et al. 2014 [50]	Suxiaojiuxin pill	HFD male apoE−/− mice	13 weeks	8 weeks	TG↓ APA↓; CPA↑ FCT↑; VEGF↓ *α*-SMA↑; MMP-2↓ MMP-9↓ TIMP-1↑ TIMP-2↑	Enhancing atherosclerotic plaque stability associated with modulating the MMPs/TIMPs balance.
Yao et al. 2014 [51]	Tongxinluo capsule	Male SD rats with the left carotid artery balloon injury	0	2 weeks	Serum ET-1↓ MCP-1↓ sICAM-1↓ NO↑; artery: ICAM-1↓ MCP-1↓; the neointimal thickening↓	Improving endothelial function, attenuating neointimal formation, and reducing inflammation.
Wang et al. 2014 [52]	Tongxinluo capsule	HFD male apoE−/− mice	12 weeks	12 weeks	TC↓ HDL↑ TG↓ LDL↓; CRP↓; APA↓; ICAM-1↓ VCAM-1↓ MCP-1↓	Preventing atherosclerotic plaque formation and intimal thickening. Reducing inflammation.
Liu et al. 2014 [53]	Danhong injection	HFD male apoE−/− mice	16 weeks	16 weeks	MCP-1↓ MMP-2↓ MMP-9↓; AAA formation↓; CPA↑	Inhibiting the high-fat diet-induced AAA formation related to the maintenance of the collagen content and the inhibition of expression of AAA-related genes.
Guo et al. 2014 [54]	Suxiaojiuxin pill	HFD male SD rats with vitamin D3	12 weeks	12 weeks	TG↓ LDL↓ TC↓ HDL↑	Reducing lipids.
Chen et al. 2014 [55]	Danhong injection	HFD male or female apoE−/− or LDLR−/− mice	16 weeks/20 weeks	16 weeks/20 weeks	Male apoE−/−: ABCA1↑ TNF-*α*↓; female apoE−/−: APA↓ LDL-C↓ HMGCR↓ LDLR↑ TNF-*α*↓; male LDLR−/−: ABCA1↑ TNF-*α*↓; female LDLR−/−: ABCA1↑ APA↓ HMGCR↓ TNF-*α*↓	Inhibiting AS through amelioration of lipid profiles.
Zhu et al. 2013 [56]	Xuezhikang	HFD male Wistar rats with vitamin D3 injection	12 weeks	12 weeks	TG↓ LDL-C↓; aorta caveolin-1↓; MDA↓ SOD↑ T-AOC↑; eNOS↑, plasma NOx↑, cGMP in erythrocyte plasma and aorta wall↑; EDI↓ blood viscosity↓	Elevating eNOS/NO, improving hemorheology, and inhibiting oxidative stress.
Zhong et al. 2013 [57]	Naoxintong capsule	HFD New Zealand rabbits	12 weeks	12 weeks	LDL-C↓ TC↓; aorta: iNOS mRNA↓ NO↓	Reducing iNOS expression in AS lesions
Zhao et al. 2013 [58]	Naoxintong capsule	HFD male LDLR−/− mice	8 weeks	8 weeks	TC↓ TG↓; APA↓; CD68↓; DCs↓ CD40↓ CD86↓ CD80↓ plasma IL-12p70↓	Protecting against AS through lipid lowering and inhibiting DCs maturation.
Li et al. 2011 [59]	Xuezhikang	HFD male Wistar rats with vitamin D3 injection	12 weeks	12 weeks	LDL-C↓ TC↓; APTT↑ PT↑ TT↑ fibrinogen↓ tissue factor↓ SOD↑ MDA↓	Inhibiting the tissue factor expression and reducing oxidative stress.
Han et al. 2011 [60]	Dahuang Zhechong pill	HFD male New Zealand rabbits with balloon injury in aorta.	60 days	60 days	Serum: MDA↓SOD↑ NO↑. Aorta: MPO↓. VSMCs: PCNA↓ Bcl-2↓	Inhibiting AS through antilipid peroxidation, protection of vascular endothelium, inhibition of VSMCs proliferation, and promotion of VSMCs apoptosis
Li et al. 2011 [61]	Suxiaojiuxin pill	HFD male SD rats with vitamin D3 injection	12 weeks	12 weeks	Serum: MDA↓ SOD↑ ox-LDL↓; PPAR *γ*↓; NF-*κ*B↓	Anti-inflammation and inhibition of oxidative stress.
Song et al. 2010 [62]	Tongxinluo capsule	HFD male Japanese rabbits	14 weeks	14 weeks	TC↓ LDL↓; PAI-1↓ VCAM-1↓	Inhibiting AS related to the reduction of blood lipid and inflammation.
Fu et al. 2009 [63]	Danhong injection	HFD male New Zealand rabbits	14 weeks	14 weeks	TC↓ TG↓ LDL-C↓; MDA↓ iNOS↓ COX-2↓; APA↓	Inhibiting AS related to the reduction of blood lipid, the inhibition of arterial wall inflammation, and the regulation of oxidative stress level.
Cao et al. 2009 [64]	Tongxinluo capsule	HFD male Japanese rabbits	14 weeks	14 weeks	APA↓; MMP-3↓ MMP-9↓ PPAR*γ*↑	Inhibiting the expression of MMP-3 and MMP-9 and increasing the expression of PPAR*γ*.
Yu et al. 2006 [65]	Tongxinluo capsule	HFD male New Zealand rabbits	16 weeks	16 weeks	APA↓; TC↓ LDL↓; macrophage↓; LOX-1↓	Inhibiting AS related to the reduction of blood lipid and LOX-1.
Xie et al. 2006 [66]	Xuezhikang	HFD Japanese rabbits	12 weeks	12 weeks	APA↓; TC↓ HDL-C↑ TG↓ LDL-C↓; serum NO↑ CRP↓	Inhibiting AS related to the reduction of blood lipid and inflammation.
Li et al. 2006 [67]	Tongxinluo capsule	HFD male Japanese rabbits with balloon injury	16 weeks	16 weeks	ET↓ NO↑; IT↓; CPA↑; MMP-1↓, COX-2↓; Bcl-2↑; FasL↓; macrophage↓	Reducing endothelial injury and intima thickness, inhibiting apoptosis, and stabilizing plaques.
Tian et al. 2004 [68]	Fufang Danshen dropping pill	HFD male New Zealand rabbits	12 weeks	12 weeks	TC↓ HDL-C↑ TG↓ LDL-C↓; IT↓	Reducing the blood lipid.
Chen et al. 2004 [69]	Fufang Danshen dropping pill	HFD male New Zealand rabbits	12 weeks	12 weeks	LO↓ APA↓; VCAM-1↓	Inhibiting VCAM-1 expression.
Guan et al. 2015 [70]	Tongxinluo capsule	Male Wistar rats with the silicone collar around the left carotid artery	0	4 weeks	pERK1/2↑ nNOS↑; LO↓	Improving the blood flow and attenuating the chronic vasoconstriction through the activation of ERK1/2 signaling.
Chen et al. 2009 [71]	Tongxinluo capsule	HFD New Zealand rabbits with balloon-induced abdominal aortic endothelial injury, undergoing plaques triggering by Chinese Russell viper venom	20 weeks	12 weeks	Serum TC↓ LDL-C↓ TG↓; MCP-1↓ hs-CRP↓ IL-8↓ IL-18↓ MMP-1↓ P-selectin↓; ultrasonography measurements: IMT↓; corrected AII ↑ APA↓ EEMA↓; MCP-1↓ MMP-1↓ MMP-3↓ MMP-12↓ P-selectin↓; vulnerability index↓ *α*-SMCs↑ CPA↓ lipid↓ RAM-11↓	Enhancing the stability of vulnerable plaques via effects on lipid lowering and anti-inflammation.
Zhang et al. 2009 [72]	Tongxinluo capsule	HFD New Zealand rabbits with balloon-induced abdominal aortic endothelial injury, undergoing an adenovirus-containing p53 and plaques triggering by Chinese Russell viper venom	10 weeks	8 weeks	Serum TC↓ LDL↓ TG↓ HDL↑; MCP-1↓ hs-CRP↓ sICAM-1↓ ox-LDL↓; ultrasonography measurements: corrected AII ↑ APA↓ EEMA↓; MCP-1↓ MMP-1↓ MMP-3↓ MMP-12↓ P-selectin↓; vulnerability index↓ *α*-SMCs↑ CPA↓ lipids↓ macrophages↓ fibrous cap thickness↓; LOX-1↓ MMP-1↓ MMP-3↓ TIMP-1↓ NF-*κ*B↓	Enhancing the stability of plaque and preventing plaque rupture via lipid lowering, anti-inflammation, and anti-oxidation.
Liu et al. 2019 [73]	Shexiang Baoxin pill	HFD LDLR−/− mice	14 weeks	14 weeks	*α*-SMA↓ SM22*α*↓ OPN↓	Reversing the dedifferentiation of VSMCs.
Meng et al. 2019 [74]	Xuezhitong capsule	HFD male apoE−/− mice	34 weeks	34 weeks	Serum TC↓ LDL↓ TG↓ HDL↑; APA↓; plasma FFA↓ ox-LDL↓ LCAT↑ ApoB↓; liver ox-LDL↓ FAS↓ LDLR↑ ABCA1↑ SR-B1↑ LCAT↑ ApoA1↑	Activating RCT and increasing HDL levels.
Gao et al. 2020 [75]	Danlou tablet	HFD male apoE−/− mice	10 weeks	10 weeks	APA↓; serum IL-8↓ MMP-1↓ MMP-2↓	Protecting against AS by reducing inflammation.
Lu et al. 2020 [76]	Guanxinshutong capsule	HFD male apoE−/− mice	10 weeks	10 weeks	Serum TC↓ LDL-C↓ TG↓ HDL-C↑; APA↓; CPA↑; CD68↓; serum TNF-*α*↓ IL-6↓ SOD↑ GSH↑ MDA↓; aortic sinus TNF-*α*↓ IL-6↓ NF-*κ*B↓ HO-1↑ Nrf2↑	Attenuating AS by reducing lipid deposition, modulating oxidative stress, and inflammatory responses
Sun et al. 2020 [77]	Danlou tablet	HFD male apoE−/− mice	32 weeks	8 weeks	Serum TC↓ TG↓ LDL-C↓; APA↓; aorta mRNA TNF-*α*↓ IL-1*β*↓ ICAM-1↓	Inhibiting AS through lipid lowering and modulating inflammation
Zhai et al. 2020 [78]	Zhixiong capsule	HFD male SD mice + vitamin D3	18 weeks	6 weeks	APA↓ IA/MA ratio↓ CPA↑ mineralization↓; serum TC↓ LDL↓ HDL↑; thoracic arteries IL-4↑ IL-13↑ MAPK1↓ MAPK14↓ p53↑	Inhibiting AS plaque progression related to the reduction of blood lipid, macrophage content, and macrophage transformation.

AAA, abdominal aortic aneurysms; ABCA1, ATP binding cassette transporter A1; ABCG1, ATP binding cassette transporter G1; ACAT, acyl coenzyme A: cholesterol acyltransferase; ANGPT-1, angiopoietin-1; APOA1, apolipoprotein A I; APOB, apolipoprotein B; AS, atherosclerosis; APA, atherosclerotic plaque area; AI, atherosclerosis index values; AII, acoustic intensities; Akt, serine/threonine kinase; AMPK, adenosine monophosphate-activated protein kinase; CHOP, CCAAT-enhancer-binding protein homologous protein; CPA, collagen-positive area; DC, dendritic cell; EDI, erythrocyte deformation index; EEMA, external elastic membrane area; FAS, fatty acid synthase; FBG, fasting blood glucose; FCA, fibrous cap area; FCT, fibrous cap thickness; FFA, free fatty acid; FINS, fasting insulin; GLUT-4, glucose transporter-4; GSH-PX, glutathione peroxidase; GSH, glutathione; HHcy, hyper-homocysteinemia; HMGCR, HMG-CoA reductase; HMGCS, HMG-CoA synthase; HO-1, heme oxygenase-1; hs-CRP, high-sensitivity C-reactive protein; ICAM-1, intercellular adhesion molecules-1; IL-6, interleukin-6; IR: insulin resistance; IA, intimal area; IT, intima thickness; IMT, intima-media thickness; LA, luminal area; LCAT, lecithin-cholesterol acyltransferase; LDH, lactate dehydrogenase; LOX-1, lectin-like oxidized low-density lipoprotein receptor-1; LO, luminal occlusion; LP-PLA2, lipoprotein-associated phospholipase A2; LXR*α*, liver X receptor *α*; MA, medial area; MDA, malondialdehyde; NADPH, nicotinamide adenine dinucleotide phosphate; NQO1, NADPH quinone oxidoreductase-1; NCA, necrotic core area; NEFA, nonesterified fatty acid; NF-*κ*B, nuclear factor-kappa B; Nrf2, nuclear factor erythroid-2-related factor 2; OPN, osteopontin; ox-LDL, oxidized low-density lipoprotein; PAI-1, plasminogen activator inhibitor 1; PI3K, phosphatidylinositol-3-kinases; PPAR*γ*, peroxisome proliferator-activated receptor *γ*; RCT, reverse cholesterol transport; SD, Sprague–Dawley; *α*-SMA, alpha smooth muscle actin; SM22*α*, smooth muscle 22 alpha; SR-B1, scavenger receptor class B type 1; SR-A1, scavenger receptor class A type 1; SOD, superoxide dismutase; sPLA2, secretory phospholipase A2; TNF-*α*, tumor necrosis factor-*α*; VV, vasa vasorum; VEGF-A, vascular endothelial growth factor-A; VCAM-1, vascular cell adhesion molecule-1; VSMCs, vascular smooth muscle cells; VEGF, vascular endothelial growth factor.

## Data Availability

All the data generated or analyzed during this study are included in this published article and its additional files.

## Abbreviations

ABCA1: ATP binding cassette transporter A1
ABCG1: ATP binding cassette transporter G1
ACAT: Acyl coenzyme A:cholesterol acyltransferase
AI: Atherosclerosis index
Akt: Serine/threonine kinase
AMPK: Adenosine monophosphate-activated protein kinase
ANGPT-1: Angiopoietin-1
APA: Atherosclerotic plaque area
APOA1: Apolipoprotein A I
APOB: Apolipoprotein B
AS: Atherosclerosis
CPA: Collagen-positive area
EEMA: External elastic membrane area
FAS: Fatty acid synthase
FCA: Fibrous cap area
FCT: Fibrous cap thickness
GSH-PX: Glutathione peroxidase
GSH: Glutathione
HMGCR: HMG-CoA reductase
HMGCS: HMG-CoA synthase
HO-1: Heme oxygenase-1
hs-CRP: High-sensitivity C-reactive protein
ICAM-1: Intercellular adhesion molecules-1
IL-6: Interleukin-6
IR: Insulin resistance
IA: Intimal area
IT: Intima thickness
IMT: Intima-media thickness
LA: Luminal area
LCAT: Lecithin-cholesterol acyltransferase
LDH: Lactate dehydrogenase
LDLR: Low-density lipoprotein receptor
LOX-1: Lectin-like oxidized low-density lipoprotein receptor-1
LO: Luminal occlusion
LP-PLA2: Lipoprotein-associated phospholipase A2
LXR*α*: Liver X receptor *α*
MA: Medial area
MDA: Malondialdehyde
NADPH: Nicotinamide adenine dinucleotide phosphate
NQO1: NADPH quinone oxidoreductase-1
NCA: Necrotic core area
NF-*κ*B: Nuclear factor-kappa B
Nrf2: Nuclear factor erythroid-2-related factor 2
ox-LDL: Oxidized low-density lipoprotein
PI3K: Phosphatidylinositol-3-kinases
PPAR*γ*: Peroxisome proliferator-activated receptor *γ*
RCT: Reverse cholesterol transport
SD: Sprague–Dawley
SREBP: Sterol regulatory element binding protein
SR-B1: Scavenger receptor class B type 1
SR-A1: Scavenger receptor class A type 1
SOD: Superoxide dismutase
sPLA2: Secretory phospholipase A2
TNF-*α*: Tumor necrosis factor-alpha
VCAM-1: Vascular cell adhesion molecule-1
VSMCs: Vascular smooth muscle cells.

## Supplementary Materials

Supplementary Materials Table S1. Classification of the postmarketing Chinese patent formulas *in vivo*. Table S2. Classification of the AS models *in vivo*. Table S3. Details of herbal drugs of the included formulas. Table S4. The bias of included animal studies according to SYRCLE's ROB tool.
