# Supplementary material for: Atheroprotective Effects and Mechanisms of Postmarketing Chinese Patent Formulas in Atherosclerosis Models: A Systematic Review
Source: Evid Based Complement Alternat Med. 2021 Nov 27;2021:4010607. doi: 10.1155/2021/4010607 (PMC8643251; doi:10.1155/2021/4010607)
Supplement: Supplementary Materials — Table S1. Classification of the postmarketing Chinese patent formulas in vivo. Table S2. Classification of the AS models in vivo. Table S3. Details of herbal drugs of the included formulas. Table S4. The bias of included animal studies according to SYRCLE's ROB tool. [file 4010607.f1.zip › 4010607.f1/Supplementary Table S1 (1).docx]

Supplementary *Table S1*: Classification of the post-marketing Chinese patent formulas *in vivo*.

| Post-marketing Chinese patent formulas | Author, year | Experimental models | HFD feeding time | | | | | Drug intervention time | | | | | Outcome measures | Effects and mechanisms |
| --- | --- | --- | --- | --- | --- | --- | --- | --- | --- | --- | --- | --- | --- | --- |
| Danhong Injection | Zhou et al. 2019 [19] | HFD male apoE-/- mice | 12 weeks | | | | | 6 weeks | | | | | TG↓HDL-C↑LDL-C↓; APA↓AI↓; NEFA↓FBG↓FINS↓IR↓; GLUT-4↑p-IRS-1↑p-AKT↑ | Attenuating AS and macrophage lipid accumulation by promoting the activation of PI3K/AKT insulin signaling pathway. |
|  | Liu et al. 2014 [53] | HFD male apoE-/- mice | 16 weeks | | | | | 16 weeks | | | | | MCP-1↓MMP-2↓MMP-9↓; AAA formation↓; CPA↑ | Inhibiting the high-fat diet-induced AAA formation related to the maintenance of the collagen content and the inhibition of expression of AAA-related genes. |
|  | Chen et al. 2014 [55] | HFD male or female apoE-/- or LDLR-/- mice | 16 weeks/20 weeks | | | | | 16 weeks/20 weeks | | | | | Male apoE-/-:ABCA1↑TNF-α↓;  female apoE-/-: APA↓ LDL-C↓ HMGCR↓ LDLR↑ TNF-α↓;  male ldlr-/-: ABCA1↑ TNF-α↓  female ldlr-/-: ABCA1↑ APA↓ HMGCR↓TNF-α↓ | Inhibiting AS through amelioration of lipid profiles. |
|  | Fu et al. 2009 [63] | HFD male New Zealand rabbits | 14 weeks | | | 14 weeks | | | | | | | TC↓ TG↓ LDL-C↓; MDA↓ iNOS↓ COX-2↓; APA↓ | Inhibiting AS related to the reduction of blood lipid, the inhibition of arterial wall inflammation and the regulation of oxidative stress level. |
| Zhixiong Capsule | Zhai et al. 2019 [20] | HFD male Japanese rabbits with the silastic collar implantation around the right carotid artery | 12 days | | | | | 12 days | | | | | TC↓HDL-C↑TC/HDL-C ratio↓log(TG/HDL-C)↓; IL-4↑; APA↓IA↓IA/MA↓ | Preventing atherosclerotic plaque formation and intimal thickening. |
|  | Zhai et al. 2020 [78] | HFD male SD mice + VitaminD3 | | | 18 weeks | | 6 weeks | | | | | APA↓ IA/MA ratio↓ CPA↑ mineralization↓; Serum TC↓LDL↓HDL↑; Thoracic arteries IL-4↑IL-13↑MAPK1↓ MAPK14↓p53↑ | | Inhibiting AS plaque progression related to the reduction of blood lipid, macrophage content and macrophage transformation. |
| Longshengzhi Capsule | Ma et al. 2019 [21] | HFD female apoE-/- mice | 18 weeks | | | | | 10 weeks | | | | | Artery sections: APA↓; MOMA2↓; NCA↓CPA↑FCA↑SMC↑; TUNEL↓; ABCA1↑ ABCG1↑.  Liver sections: lipid droplets↓ Liver TG↓; FA oxidation↑ FA synyhesis↓ SREBP1↓; SREBP2↓ LDLR↑ HMGCS↓; DGAT1↓ ATGL↑ MTTP↓ APOC2↑; CCR2↓ IL-6↓ MCP-1↓ TNF-α↓; CD68↓ MOMA2↓.  Serum TNF-α↓ | Reducing AS by reducing macrophage/foam cell accumulation, maintaining the integrity of arterial wall, ameliorating hepatic lipid metabolism and inhibiting inflammation. |
| Tongxinluo Capsule | Ma et al. 2019 [22] | HFD male apoE-/- mice | 16 weeks | | | | | 16 weeks | | | | | APA↓; CPA↑ SMC↑ Staining of lipids and macrophages↓; IL-6↓, MMP2↓ and TNF-α↓ | Inhibiting AS development and stabilizing plaque. |
|  | Yin et al. 2018 [27] | HFD male and female New Zealand rabbits with the silicone tube encapsulation of left carotid artery | | 4 weeks | | | | | | 4 weeks | | | TC↓ TG↓ LDL-C↓; Serum MDA↓ SOD↑, and T-AOC↑; VEGF-A↓ VEGF-R2↓; Nuclear NF-κB↓ TNF-α↓ IL-6↓; Nuclear Nrf2↑ NQO1↑ | Reducing carotid adventitial VV angiogenesis and alleviating early AS lesions by inhibiting carotid inflammation and oxidative stress injury. |
|  | Chen et al. 2018 [31] | HFD male apoE-/- mice with silastic collar implantation | 8 weeks | | | | | 8 weeks | | | | | TUNEL↓Lc3b dots↑; APA↓; Vulnerable index↓ macrophage apoptosis↓ | Improving autophagy via Beclin-1. |
|  | Ma et al. 2016 [41] | HFD male apoE-/- mice | 5 weeks | | | | | 5 weeks | | | | | VEGF-A↓ ANGPT-1↑; microvessels sprouting↓ VV number in plaques↓; APA↓; CPA↑ SMC↑ MOMA2↓ FCT↑ | Inhibiting early AS through regulating angiogenic factor expression and inhibiting VV proliferation in atherosclerotic plaque. |
|  | Wu et al. 2015 [45] | HFD male apoE-/- mice | 12 weeks | | | | | 12 weeks | | | | | APA↓; p22↓p47↓HO-1↓; NF-κB↓; TC↓ TG↓ LDL↓ | Decreasing atherosclerotic plaque formation and inhibiting oxidative stress and inflammation. |
|  | Lang et al. 2015 [46] | HFD New Zealand rabbits with the silastic collar implantation around the right carotid artery | 4 weeks | | | | | 4 weeks | | | | | IT↓; CD34↓; Microvascular blood flow volume↓；VAGF↓ VEGFR-2↓ | Inhibiting VV proliferation. |
|  | Zhang et al. 2014 [49] | Male C57BL/6 mice with the left common carotid artery ligation | 0 | | | | | 21 days | | | | | IA/MA ratio↓IA↓; TNF-α↓IL-1β↓; miR-155↓ | Inhibiting the vascular inflammatory response and neointimal hyperplasia. |
|  | Yao et al. 2014 [51] | Male SD rats with the left carotid artery balloon injury | 0 | | | | | 2 weeks | | | | | Serum ET-1↓MCP-1↓sICAM-1↓NO↑; Artery: ICAM-1↓MCP-1↓; the neointimal thickening↓ | Improving endothelial function, attenuating neointimal formation, and reducing inflammation. |
|  | Wang et al. 2014 [52] | HFD male apoE-/- mice | 12 weeks | | | | | 12 weeks | | | | | TC↓ HDL↑ TG↓ LDL↓; CRP↓; APA↓; ICAM-1↓ VCAM-1↓ MCP-1↓ | Preventing atherosclerotic plaque formation and intimal thickening.  Reducing inflammation. |
|  | Song et al. 2010 [62] | HFD male Japanese rabbits | 14 weeks | | | | | 14 weeks | | | | | TC↓ LDL↓; PAI-1↓ VCAM-1↓ | Inhibiting AS related to the reduction of blood lipid and inflammation. |
|  | Cao et al. 2009 [64] | HFD male Japanese rabbits | 14 weeks | | | | | 14 weeks | | | | | APA↓; MMP-3↓ MMP-9↓ PPARγ↑ | Inhibiting the expression of MMP-3 and MMP-9 and increasing the expression of PPARγ. |
|  | Yu et al. 2006 [65] | HFD male New Zealand rabbits | 16 weeks | | | | | 16 weeks | | | | | APA↓; TC↓ LDL↓; Macrophage↓; LOX-1↓ | Inhibiting AS related to the reduction of blood lipid and LOX-1. |
|  | Li et al. 2006 [67] | HFD male Japanese rabbits with balloon injury | 16 weeks | | | | | 16 weeks | | | | | ET↓ NO↑; IT↓; CPA↑; MMP-1↓, COX-2↓; Bcl-2↑; FasL↓; Macrophage↓ | Reducing endothelial injury and intima thickness, inhibiting apoptosis and stabilizing plaques. |
|  | Guan et al. 2015 [70] | Male Wistar rats with the silicone collar around the left carotid artery | | 0 | | | | | 4 weeks | | | | pERK1/2↑ nNOS↑; LO↓ | Improving the blood flow and attenuating the chronic vasoconstriction through activation of ERK1/2 signaling. |
|  | Chen et al. 2009 [71] | HFD New Zealand rabbits with balloon-induced abdominal aortic endothelial injury, undergoing plaques triggering by Chinese Russell viper venom | | 20 weeks | | | | | | | 12 weeks | | Serum TC↓ LDL-C↓ TG↓; MCP-1↓ hs-CRP↓ IL-8↓ IL-18↓ MMP-1↓ P-selectin↓; Ultrasonography measurements: IMT↓; corrected AII ↑ APA↓ EEMA↓; MCP-1↓ MMP-1↓ MMP-3↓ MMP-12↓ P-selectin↓; vulnerability index↓ α-SMCs↑ CPA↓ Lipid↓ RAM-11↓ | Enhancing the stability of vulnerable plaques via effects on lipid lowering and anti-inflammation. |
|  | Zhang et al. 2009 [72] | HFD New Zealand rabbits with balloon-induced abdominal aortic endothelial injury, undergoing an adenovirus containing p53 and plaques triggering by Chinese Russell viper venom | | 10 weeks | | | | | | | 8 weeks | | Serum TC↓ LDL↓ TG↓ HDL↑; MCP-1↓ hs-CRP↓ sICAM-1↓ ox-LDL↓; Ultrasonography measurements: corrected AII ↑ APA↓ EEMA↓; MCP-1↓ MMP-1↓ MMP-3↓ MMP-12↓ P-selectin↓; vulnerability index↓ α-SMCs↑ CPA↓ Lipids↓ Macrophages↓ Fibrous Cap Thickness↓; LOX-1↓ MMP-1↓ MMP-3↓ TIMP-1↓ NF-κB↓ | Enhancing the stability of plaque and preventing plaque rupture via lipid lowering, anti-inflammation and anti-oxidation. |
| Shexiang Baoxin Pill | Lu et al. 2019 [23] | HFD apoE-/- mice | 20 weeks | | | | | 20 weeks | | | | | APA↓; SOD↑ CAT↑ GSH↑ MDA↓ H2O2↓ and MPO↓; MCP-1↓ IFN-γ↓ IL-17A↓ IL-10 ↑ TGF-β1↑; VCAM-1↓ ICAM-1↓ IL-6↓ IL-2↓; macrophages↓ ABCA1↑ ABCG1↑; p38↓ JNK↓ Mfn2↑ NF-κB↓ SR-A↓ LOX-1↓ LXRα↑ | Exerting anti-atherosclerotic effects via improving inflammation response and inhibiting lipid accumulation. |
|  | Liu et al. 2019 [73] | HFD LDLR-/- mice | 14 weeks | | | | | 14 weeks | | | | | α-SMA↓ SM22α↓ OPN↓ | Reversing the dedifferentiation of VSMCs |
| Danlou Tablet | Hao et al. 2019 [24] | HFD male apoE-/- mice | 20 weeks | | | | | 12 weeks | | | | | APA↓; LO↓; Lipid content in artery↓ HDL-C↑ ox-LDL↓; IL-1β↓, IL-10↓, MCP-1↓, IL-18 ↓, IL-33↓; PPARα↑, PGC-1α↑, ABCA1↑, P-IKKα/β↓, P-IκBα↓ and P-NF-κBp65↓ | Preventing AS via suppressing NF-κB signaling and triggering PPARα/ABCA1 signaling pathway. |
|  | Chen et al. 2016 [42] | HFD male Wistar rats with Vitamin D3 injection | 4 weeks | | | | | 8 weeks | | | | | TC↓ TG↓ LDL-C↓; APA↓; IL-6↓ TNF-α↓ MCP-1↓ ox-LDL↓;LP-PLA2↓ sPLA2↓ | Inhibiting AS related to the reduction of blood lipid and inflammation. |
|  | Gao et al. 2020 [75] | HFD male apoE-/- mice | 10 weeks | | | | | 10 weeks | | | | | APA↓; Serum IL-8↓MMP-1↓MMP-2↓ | Protecting against AS by reducing inflammation |
|  | Sun et al. 2020 [77] | HFD male apoE-/- mice | 32 weeks | | | | | 8 weeks | | | | | Serum TC↓TG↓LDL-C↓; APA↓; Aorta mRNA TNF-α↓IL-1β↓ICAM-1↓ | Inhibiting AS through lipid-lowering and modulating inflammation |
| Danlou Tablet and Xuefu Zhuyu Granule | Miao et al. 2016 [40] | HFD male Wistar rats with Vitamin D3 injection | 4 weeks | | | | | 8 weeks | | | | | Danlou group: IT↓ TC↓ TG↓ LDL-C↓ PDGF↓ ERK1/2↓ pERK1/2↓.  Xuefu Zhuyu group: IT↓ TC↓ PDGF↓ ERK1/2↓ pERK1/2↓ | Reducing serum lipids and increasing PDGF, inhibiting ERK signal pathway activation and VSMC proliferation. |
| Angong Niuhuang Pill | Chai et al. 2019 [25] | HFD male apoE-/- mice | 8 weeks | | | | | 8 weeks | | | | | Aorta: MCP-1↓ MCP-2↓ MCP-3↓ CCR2↓ CXCR3↓; ICAM-1↓ VCAM-1↓; IL-6↓ TGF-β1↑ IL-17↓; Treg cell↑; Th17/ Treg cell↓.  Spleen: IL-6↓ TGF-β1↑ | Ameliorating the development of early AS by reducing splenic and vascular inflammation. |
|  | Fu et al. 2017 [35] | HFD male SD rats with Vitamin D3 injection | 17 weeks | | | | | 9 weeks | | | | | APA↓IT↓MT↓, the maximum platelet aggregation rates↓; serum TC↓ LDL-C↓ TC/HDL-C↓ LDL-C/HDL-C↓; MDA↓ hsCRP↓ LDH↓ cTnI↓; Myocardial fibers↓; Bax protein↓ Bcl-2↑ | Reducing AS due to its anti-platelet aggregation, lipid regulatory, antioxidant, anti-inflammatory and anti-apoptotic properties. |
| Longxuetongluo Capsule | Zhou et al. 2018 [26] | HFD male SD rats | 4 weeks | | | | | 4 weeks | | | | | TC↓ HDL-C↑ LDL-C↓ TG↓; Serum ALT↓ AST↓ Serum MCP-1↓ ICAM-1↓ VCAM-1↓.  Histological sections of liver and aorta↓; Aortic histological sections: NF-κB↓ | Preventing AS and fatty liver by controlling lipid metabolism and anti-inflammation activity. |
|  | Zheng et al. 2016 [38] | HFD male apoE-/- mice | 6 weeks | | | | | 6 weeks | | | | | APA↓ | Reducing plaques. |
| Longhu Rendan | Yan et al. 2018 [28] | HFD male apoE-/- mice | 10 weeks | | | | | 10 weeks | | | | | TC↓LDL-C↓TG↓; APA↓; LOX-1↓ | Ameliorating AS via reducing serum lipid and LOX-1 expression. |
| Naoxintong Pill | Wang et al. 2018 [29] | HFD male apoE-/- mice | 8 weeks | | | | | 8 weeks | | | | | Plaque foam cell content↓ | Reducing foam cell accumulation in atherosclerotic plaques. |
| Naoxintong Capsule | Yang et al. 2017 [32] | HFD male apoE-/- mice | 18 weeks | | | | | 8 weeks | | | | | APA↓LO↓; HDL-C↑; CPA↑ SMC↑ MPO↑ CD68↓ Calcification events↓ Fibrous cap thickness↑.  Liver sections: SREBP1↑ and SREBP2↑; ATGL↑ and LDLR↑; Liver TG↓; DGAT1↓CGI-58↑; ATGL↑ pi-AMPKa↑ | Inhibiting AS development, stabilizing plaque and reducing hepatic triglyceride levels. |
|  | Yang et al. 2016 [39] | HFD male apoE-/- mice | 18 weeks | | | | | 8 weeks | | | | | APA↓ CPA↑ SMC↑ Calcification events↓ Fibrous cap thickness↑ MOMA2 protein↓ MMP-2↓ and TNFα↓ SM22α↑ | Reducing advanced AS and enhancing the plaque stability. |
|  | Zhong et al. 2013 [57] | HFD New Zealand rabbits | 12 weeks | | | | | 12 weeks | | | | | LDL-C↓ TC↓; Aorta: iNOS mRNA↓ NO↓ | Reducing iNOS expression in AS lesions |
|  | Zhao et al. 2013 [58] | HFD male LDLR-/- mice | 8 weeks | | | | | 8 weeks | | | | | TC↓TG↓; APA↓; CD68↓; DCs↓ CD40↓ CD86↓ CD80↓ plasma IL-12p70↓ | Protecting against AS through lipid-lowering and inhibiting DCs maturation. |
| Di'ao Xinxuekang Capsule | Qu et al. 2018 [30] | HFD male apoE-/- mice | 18 weeks | | | | | 18 weeks | | | | | TC↓HDL-C↑LDL-C↓TG↓; APA↓.  Liver sections: lipid accumulation↓; PCSK9↓; liver LDLR↑  serum PCSK9↓ | Alleviating lipid disorder and ameliorating AS with down-regulation of the PCSK9. |
|  | Dong et al. 2017 [36] | HFD male apoE-/- mice | 8 weeks | | | | | 8 weeks | | | | | TC↓ LDL-C↓; APA↓.  Aorta sections: ABCA1↑ and ABCG1↑  Liver and intestines: ABCA1↑ ApoA-I↑ PPARγ↑ LXRα↑  Liver sections: SR-B1↑ preB1-HDL↓ HDL3↓ HDL2↑.  Serum LCAT↑ | Regulating RCT by improving HDL synthesis, maturation and catabolism. |
| Xuezhikang | Shen et al. 2017 [33] | Female apoE-/- mice combined partial ligation of the left common carotid artery and left renal artery | 0 | | | | | 8 weeks | | | | | APA↓; CD68↓ α-SMA↑ CPA↑ Vulnerable Phenotype↓; p-PERK↓ p-IRE1α↓ p-eIF2α↓ and BiP↓ CHOP↓ DHE staining↓; NCA↓ TUNEL↓ caspase-3↓; TNFα↓ MMP8↓ and MMP13↓ | Suppressing vulnerable plaque progression and rupture by mitigating lesional endoplasmic reticulum stress and inhibiting apoptosis and the NF-κB pro-inflammatory pathway. |
|  | Zhu et al. 2013 [56] | HFDmale Wistar rats with Vitamin D3 injection | 12 weeks | | | | | 12 weeks | | | | | TG↓ LDL-C↓; Aorta caveolin-1↓; MDA↓ SOD↑ and T-AOC↑; eNOS↑, plasma NOx↑, cGMP in erythrocyte plasma and aorta wall↑; EDI↓ blood viscosity↓ | Elevating eNOS/NO, improving hemorheology and inhibiting oxidative stress. |
|  | Li et al. 2011 [59] | HFDmale Wistar rats with Vitamin D3 injection | 12 weeks | | | | | 12 weeks | | | | | LDL-C↓ TC↓; APTT↑ PT↑ TT↑ Fibrinogen↓ tissue factor↓SOD↑ MDA↓ | Inhibiting the tissue factor expression and reducing oxidative stress. |
|  | Xie et al. 2006 [66] | HFD Japanese rabbits | 12 weeks | | | | | 12 weeks | | | | | APA↓; TC↓ HDL-C↑ TG↓ LDL-C↓; Serum NO↑ CRP↓ | Inhibiting AS related to the reduction of blood lipid and inflammation. |
| Qishenyiqi Pill | Peng et al. 2017 [34] | HFD male apoE-/- mice | 8 weeks | | | | | 8 weeks | | | | | APA↓LDL-C↓ Liver weight/Body weight↓  Liver sections: LXRα↑ABCG5↑  Aorta sections: CD36↓ Foxp3↑ IL-17A↓  Spleen sections: Foxp3↓ IL-17A↓ Smad2/3↓ IL-6↓ RORγ↓ | Promoting regulatory T cells in atherosclerotic lesion, inhibiting T helper 17 cells in plaque and spleen and accelerating liver cholesterol excretion. |
| Ginkgo Biloba Tablet | Zhu et al. 2016 [37] | HFD male Wistar rats with Vitamin D3 injection and balloon injury in aorta. | 60 days | | | | | 60 days | | | | | Blood glucose and calcium↓; TC↓ TG↓ LDL-C↓; LO↓ IT↓; SR-A↓; CRP↓ ICAM-1↓ VCAM-1↓ | Alleviating AS lesions by inhibiting inflammation and controlling lipid. |
| Shexiang Tongxin Dropping Pill | Xiong et al. 2015 [43] | HFD male apoE-/- mice | 8 weeks | | | | | 8 weeks | | | | | IL-2↓ IL-6↓ TNF-α↓ INF-γ↓ ox-LDL↓ MDA↓; GSH↑ SOD↑; ROS↓;miR-21↓ miR-126↓ miR-155↓, miR-20↑ | Inhibiting AS via reducing inflammation and regulating miR-21, miR-126, miR-155 and miR-20. |
|  | Xiong et al. 2015 [44] | HFD male apoE-/- mice | 8 weeks | | | | | 8 weeks | | | | | APA↓; TC↓ TG↓ LDL↓ ox-LDL↓ HDL↑; IL-2↓ IL-6↓ TNF-α↓ INF-γ↓ ox-LDL↓ MDA↓; GSH↑ SOD↑ ROS↓; miR-21↓ miR-126↓ miR-155↓ miR-132↓ miR-20↑ | Inhibiting AS via reducing inflammation and regulating miR-21, miR-126, miR-155, miR-132 and miR-20. |
| Compound Chuanxiong Capsule | Kang et al. 2015 [47] | HFD male apoE-/- mice | 13 weeks | | | | | 7 weeks | | | | | TC↓ TG↓ LDL-C↓; AI↓ APA↓; CPA↑; PI3K↓ Akt↓ NF-kB↓ IL-6↓TNF-α↓ | Preventing AS and inhibiting the expression of IL-6 and TNF-𝛼 by regulating PI3K/Akt/NF-𝜅B signaling pathway. |
| Yindanxinnaotong Soft Capsule | Cheng et al. 2015 [48] | HFD male SD rats with Vitamin D3 injection | 9 weeks | | | | | 12 weeks | | | | | APA↓TC↓TG↓LDL-C↓; MDA↓SOD↑GSH↑GSH-px↑; NF-kB↓ IkB↑; IL-1β↓ CRP↓ TNF-α↓; NO↑ TXB2↓ | Relieving AS through regulating lipids, reducing lipid particle deposition in the endothelial layer of artery, enhancing antioxidant power, and repressing inflammation activity by inhibiting NF-κB signal pathway. |
| Suxiaojiuxin Pill | Zhang et al. 2014 [50] | HFD male apoE-/- mice | 13 weeks | | | | | 8 weeks | | | | | TG↓ APA↓; CPA↑ FCT↑; VEGF↓ α-SMA↑; MMP-2↓MMP-9↓TIMP-1↑ TIMP-2↑ | Enhancing atherosclerotic plaque stability associated with modulating the MMPs/TIMPs balance. |
|  | Guo et al. 2014 [54] | HFD male SD rats with Vitamin D3 | 12 weeks | | | | | 12 weeks | | | | | TG↓ LDL↓ TC↓ HDL↑ | Reducing lipids. |
|  | Li et al. 2011 [61] | HFDmale SD rats with Vitamin D3 injection | 12 weeks | | | | | 12 weeks | | | | | Serum: MDA↓ SOD↑ ox-LDL↓;  PPAR γ↓; NF-κB↓ | Anti-inflammation and inhibiting of oxidative stress, |
| Dahuang Zhechong Pill | Han et al. 2011 [60] | HFD male New Zealand rabbits with balloon injury in aorta. | 60 days | | | | | 60 days | | | | | Serum: MDA↓SOD↑ NO↑  Aorta: MPO↓ VSMCs: PCNA↓ Bcl-2↓ | Inhibiting AS through anti-lipid peroxidation, protection of vascular endothelium, inhibition of VSMCs proliferation and promotion of VSMCs apoptosis |
| Fufang Danshen Dropping Pill | Tian et al. 2004 [68] | HFD male New Zealand rabbits | 12 weeks | | | | | 12 weeks | | | | | TC↓ HDL-C↑ TG↓ LDL-C↓; IT↓ | Reducing the blood lipid. |
|  | Chen et al. 2004 [69] | HFD male New Zealand rabbits | 12 weeks | | | | | 12 weeks | | | | | LO↓ APA↓; VCAM-1↓ | Inhibiting VCAM-1 expression. |
| Xuezhitong Capsule | Meng et al. 2019 [74] | HFD male apoE-/- mice | 34 weeks | | | | | 34 weeks | | | | | Serum TC↓ LDL↓ TG↓ HDL↑; APA↓; Plasma FFA↓ ox-LDL↓ LCAT↑ ApoB↓; Liver ox-LDL↓ FAS↓ LDLR↑ ABCA1↑ SR-B1↑ LCAT↑ ApoA1↑ | Activating RCT and increasing HDL levels |
| Guanxinshutong capsule | Lu et al. 2020 [76] | HFD male apoE-/- mice | 10 weeks | | | | | 10 weeks | | | | | Serum TC↓LDL-C↓TG↓HDL-C↑; APA↓; CPA↑; CD68↓; Serum TNFα↓ IL-6↓SOD↑GSH↑MDA↓; Aortic sinus TNFα↓IL-6↓NF-κB↓HO-1↑Nrf2↑ | Attenuating AS by reducing lipid deposition, modulating oxidative stress and inflammatory responses |

AAA, abdominal aortic aneurysms; ABCA1, ATP binding cassette transporter A1; ABCG1, ATP binding cassette transporter G1; ACAT, acyl coenzyme A: cholesterol acyltransferase; ANGPT-1, angiopoietin-1; APOA1, apolipoprotein A I; APOB, apolipoprotein B; AS, atherosclerosis; APA, atherosclerotic plaque area; AI, atherosclerosis index values; AII, acoustic intensities; Akt, serine/threonine kinase; AMPK, adenosine monophosphate-activated protein kinase; CHOP, CCAAT-enhancer-binding protein homologous protein; CPA, collagen positive area; DC, dendritic cell; EDI, erythrocyte deformation index; EEMA, external elastic membrane area; FAS, fatty acid synthase; FBG, fasting blood glucose; FCA, fibrous cap area; FCT, fibrous cap thickness; FFA, free fatty acid; FINS, fasting insulin; GLUT-4, glucose transporter-4; GSH-PX, glutathione peroxidase; GSH, glutathione; HHcy, hyper-homocysteinemia; HMGCR, HMG-CoA reductase; HMGCS, HMG-CoA synthase; HO-1, heme oxygenase-1; hs-CRP, high-sensitivity C-reactive protein; ICAM-1, intercellular adhesion molecules-1; IL-6, interleukin-6; IR: insulin resistance; IA, intimal area; IT, intima thickness; IMT, intima–media thickness; LA, luminal area; LCAT, lecithin-cholesterol acyltransferase; LDH, lactate dehydrogenase; LOX-1, lectin-like oxidized low-density-lipoprotein receptor-1; LO, luminal occlusion; LP-PLA2, lipoprotein-associated phospholipase A2; LXRα, liver X receptor α; MA, medial area; MDA, malondialdehyde; NADPH, nicotinamide adenine dinucleotide phosphate; NQO1, NADPH quinone oxidoreductase 1; NCA, necrotic core area; NEFA, non-esterified fatty acid; NF-𝜅B, nuclear factor-kappa B; Nrf2, nuclear factor erythroid-2-related factor 2; OPN, osteopontin; ox-LDL, oxidized low-density lipoprotein; PAI-1, plasminogen activator inhibitor 1; PI3K, phosphatidylinositol-3-kinases; PPARγ, peroxisome proliferator-activated receptor γ; RCT, reverse cholesterol transport; SD, Sprague-Dawley; α-SMA, alpha smooth muscle actin; SM22α, smooth muscle 22 alpha; SR-B1, scavenger receptor class B type 1; SR-A1, scavenger receptor class A type 1; SOD, superoxide dismutase; sPLA2, secretory phospholipase A2; TNF-𝛼, tumor necrosis factor-𝛼; VV, vasa vasorum; VEGF-A, vascular endothelial growth factor A; VCAM-1, vascular cell adhesion molecule 1; VSMCs, vascular smooth muscle cells; VEGF, vascular endothelial growth factor.
