# Supplementary material for: Atheroprotective Effects and Mechanisms of Postmarketing Chinese Patent Formulas in Atherosclerosis Models: A Systematic Review
Source: Evid Based Complement Alternat Med. 2021 Nov 27;2021:4010607. doi: 10.1155/2021/4010607 (PMC8643251; doi:10.1155/2021/4010607)
Supplement: Supplementary Materials — Table S1. Classification of the postmarketing Chinese patent formulas in vivo. Table S2. Classification of the AS models in vivo. Table S3. Details of herbal drugs of the included formulas. Table S4. The bias of included animal studies according to SYRCLE's ROB tool. [file 4010607.f1.zip › 4010607.f1/Supplementary Table S2 (1).docx]

Supplementary *Table S2*: Classification of the AS models *in vivo*.

| Experimental models | Author, year | Post-marketing Chinese patent formulas | HFD feeding time | Drug intervention time | | | | | Outcome measures | | | | | | | Effects and mechanisms | |
| --- | --- | --- | --- | --- | --- | --- | --- | --- | --- | --- | --- | --- | --- | --- | --- | --- | --- |
| HFD male apoE-/- mice | Zhou et al. 2019 [19] | Danhong Injection | 12 weeks | 6 weeks | | | | | TG↓HDL-C↑LDL-C↓; APA↓AI↓; NEFA↓FBG↓FINS↓IR↓; GLUT-4↑p-IRS-1↑p-AKT↑ | | | | | | | Attenuating AS and macrophage lipid accumulation by promoting the activation of PI3K/AKT insulin signaling pathway. | |
| HFD male apoE-/- mice | Ma et al. 2019 [22] | Tongxinluo Capsule | 16 weeks | 16 weeks | | | | | APA↓; CPA↑ SMC↑ Staining of lipids and macrophages↓; IL-6↓, MMP2↓ and TNF-α↓ | | | | | | | Inhibiting AS development and stabilizing plaque. | |
| HFD male apoE-/- mice | Hao et al. 2019 [24] | Danlou Tablet | 20 weeks | 12 weeks | | | | | APA↓; LO↓; Lipid content in artery↓ HDL-C↑ ox-LDL↓; IL-1β↓, IL-10↓, MCP-1↓, IL-18 ↓, IL-33↓; PPARα↑, PGC-1α↑, ABCA1↑, P-IKKα/β↓, P-IκBα↓ and P-NF-κBp65↓ | | | | | | | Preventing AS via suppressing NF-κB signaling and triggering PPARα/ABCA1 signaling pathway. | |
| HFD male apoE-/- mice | Chai et al. 2019 [25] | Angong Niuhuang Pill | 8 weeks | 8 weeks | | | | | Aorta: MCP-1↓ MCP-2↓ MCP-3↓ CCR2↓ CXCR3↓; ICAM-1↓ VCAM-1↓; IL-6↓ TGF-β1↑ IL-17↓; Treg cell↑; Th17/ Treg cell↓.  Spleen: IL-6↓ TGF-β1↑ | | | | | | | Ameliorating the development of early AS by reducing splenic and vascular inflammation. | |
| HFD male apoE-/- mice | Yan et al. 2018 [28] | Longhu Rendan | 10 weeks | 10 weeks | | | | | TC↓LDL-C↓TG↓; APA↓; LOX-1↓ | | | | | | | Ameliorating AS via reducing serum lipid and LOX-1 expression. | |
| HFD male apoE-/- mice | Wang et al. 2018 [29] | Naoxintong Pill | 8 weeks | 8 weeks | | | | | Plaque foam cell content↓ | | | | | | | Reducing foam cell accumulation in atherosclerotic plaques. | |
| HFD male apoE-/- mice | Qu et al. 2018 [30] | Di'ao Xinxuekang Capsule | 18 weeks | 18 weeks | | | | | TC↓HDL-C↑LDL-C↓TG↓; APA↓.  Liver sections: lipid accumulation↓; PCSK9↓; liver LDLR↑  serum PCSK9↓ | | | | | | | Alleviating lipid disorder and ameliorating AS with down-regulation of the PCSK9. | |
| HFD male apoE-/- mice | Yang et al. 2017 [32] | Naoxintong Capsule | 18 weeks | 8 weeks | | | | | APA↓LO↓; HDL-C↑; CPA↑ SMC↑ MPO↑ CD68↓ Calcification events↓ Fibrous cap thickness↑.  Liver sections: SREBP1↑ and SREBP2↑; ATGL↑ and LDLR↑; Liver TG↓; DGAT1↓CGI-58↑; ATGL↑ pi-AMPKa↑ | | | | | | | Inhibiting AS development, stabilizing plaque and reducing hepatic triglyceride levels. | |
| HFD male apoE-/- mice | Peng et al. 2017 [34] | Qishenyiqi Pill | 8 weeks | 8 weeks | | | | | APA↓LDL-C↓ Liver weight/Body weight↓  Liver sections: LXRα↑ABCG5↑  Aorta sections: CD36↓ Foxp3↑ IL-17A↓  Spleen sections: Foxp3↓ IL-17A↓ Smad2/3↓ IL-6↓ RORγ↓ | | | | | | | Promoting regulatory T cells in atherosclerotic lesion, inhibiting T helper 17 cells in plaque and spleen and accelerating liver cholesterol excretion. | |
| HFD male apoE-/- mice | Dong et al. 2017 [36] | Di'ao Xinxuekang Capsule | 8 weeks | 8 weeks | | | | | TC↓ LDL-C↓; APA↓.  Aorta sections: ABCA1↑ and ABCG1↑  Liver and intestines: ABCA1↑ ApoA-I↑ PPARγ↑ LXRα↑  Liver sections: SR-B1↑ preB1-HDL↓ HDL3↓ HDL2↑.  Serum LCAT↑ | | | | | | | Regulating RCT by improving HDL synthesis, maturation and catabolism. | |
| HFD male apoE-/- mice | Zheng et al. 2016 [38] | Longxuetongluo Capsule | 6 weeks | 6 weeks | | | | | APA↓ | | | | | | | Reducing plaques. | |
| HFD male apoE-/- mice | Yang et al. 2016 [39] | Naoxintong Capsule | 18 weeks | 8 weeks | | | | | APA↓ CPA↑ SMC↑ Calcification events↓ Fibrous cap thickness↑ MOMA2 protein↓ MMP-2↓ and TNFα↓ SM22α↑ | | | | | | | Reducing advanced AS and enhancing the plaque stability. | |
| HFD male apoE-/- mice | Xiong et al. 2015 [43] | Shexiang Tongxin Dropping Pill | 8 weeks | 8 weeks | | | | | IL-2↓ IL-6↓ TNF-α↓ INF-γ↓ ox-LDL↓ MDA↓; GSH↑ SOD↑; ROS↓;miR-21↓ miR-126↓ miR-155↓, miR-20↑ | | | | | | | Inhibiting AS via reducing inflammation and regulating miR-21, miR-126, miR-155 and miR-20. | |
| HFD male apoE-/- mice | Xiong et al. 2015 [44] | Shexiang Tongxin Dropping Pill | 8 weeks | 8 weeks | | | | | APA↓; TC↓ TG↓ LDL↓ ox-LDL↓ HDL↑; IL-2↓ IL-6↓ TNF-α↓ INF-γ↓ ox-LDL↓ MDA↓; GSH↑ SOD↑ ROS↓; miR-21↓ miR-126↓ miR-155↓ miR-132↓ miR-20↑ | | | | | | | Inhibiting AS via reducing inflammation and regulating miR-21, miR-126, miR-155, miR-132 and miR-20. | |
| HFD male apoE-/- mice | Wu et al. 2015 [45] | Tongxinluo Capsule | 12 weeks | 12 weeks | | | | | APA↓; p22↓p47↓HO-1↓; NF-κB↓; TC↓ TG↓ LDL↓ | | | | | | | Decreasing atherosclerotic plaque formation and inhibiting oxidative stress and inflammation. | |
| HFD male apoE-/- mice | Ma et al. 2016 [41] | Tongxinluo Capsule | 5 weeks | 5 weeks | | | | | VEGF-A↓ ANGPT-1↑; microvessels sprouting↓ VV number in plaques↓; APA↓; CPA↑ SMC↑ MOMA2↓ FCT↑ | | | | | | | Inhibiting early AS through regulating angiogenic factor expression and inhibiting VV proliferation in atherosclerotic plaque. | |
| HFD male apoE-/- mice | Zhang et al. 2014 [50] | Suxiaojiuxin Pill | 13 weeks | 8 weeks | | | | | TG↓ APA↓; CPA↑ FCT↑; VEGF↓ α-SMA↑; MMP-2↓MMP-9↓TIMP-1↑ TIMP-2↑ | | | | | | | Enhancing atherosclerotic plaque stability associated with modulating the MMPs/TIMPs balance. | |
| HFD male apoE-/- mice | Kang et al. 2015 [47] | Compound Chuanxiong Capsule | 13 weeks | 7 weeks | | | | | TC↓ TG↓ LDL-C↓; AI↓ APA↓; CPA↑; PI3K↓ Akt↓ NF-kB↓ IL-6↓TNF-α↓ | | | | | | | Preventing AS and inhibiting the expression of IL-6 and TNF-𝛼 by regulating PI3K/Akt/NF-𝜅B signaling pathway. | |
| HFD male apoE-/- mice | Wang et al. 2014 [52] | Tongxinluo Capsule | 12 weeks | 12 weeks | | | | | TC↓ HDL↑ TG↓ LDL↓; CRP↓; APA↓; ICAM-1↓ VCAM-1↓ MCP-1↓ | | | | | | | Preventing atherosclerotic plaque formation and intimal thickening.  Reducing inflammation. | |
| HFD male apoE-/- mice | Liu et al. 2014 [53] | Danhong Injection | 16 weeks | 16 weeks | | | | | MCP-1↓MMP-2↓MMP-9↓; AAA formation↓; CPA↑ | | | | | | | Inhibiting the high-fat diet-induced AAA formation related to the maintenance of the collagen content and the inhibition of expression of AAA-related genes. | |
| HFD male apoE-/- mice | Meng et al. 2019 [74] | Xuezhitong Capsule | 34 weeks | 34 weeks | | | | | Serum TC↓ LDL↓ TG↓ HDL↑; APA↓; Plasma FFA↓ ox-LDL↓ LCAT↑ ApoB↓; Liver ox-LDL↓ FAS↓ LDLR↑ ABCA1↑ SR-B1↑ LCAT↑ ApoA1↑ | | | | | | | Activating RCT and increasing HDL levels | |
| HFD male apoE-/- mice | Gao et al. 2020 [75] | Danlou Tablet | 10 weeks | 10 weeks | | | | | APA↓; Serum IL-8↓MMP-1↓MMP-2↓ | | | | | | | Protecting against AS by reducing inflammation | |
| HFD male apoE-/- mice | Lu et al. 2020 [76] | Guanxinshutong capsule | 10 weeks | 10 weeks | | | | | Serum TC↓LDL-C↓TG↓HDL-C↑; APA↓; CPA↑; CD68↓; Serum TNFα↓ IL-6↓SOD↑GSH↑MDA↓; Aortic sinus TNFα↓IL-6↓NF-κB↓HO-1↑Nrf2↑ | | | | | | | Attenuating AS by reducing lipid deposition, modulating oxidative stress and inflammatory responses | |
| HFD male apoE-/- mice | Sun et al. 2020 [77] | Danlou Tablet | 32 weeks | 8 weeks | | | | | Serum TC↓TG↓LDL-C↓; APA↓; Aorta mRNA TNF-α↓IL-1β↓ICAM-1↓ | | | | | | | Inhibiting AS through lipid-lowering and modulating inflammation | |
| HFD female apoE-/- mice | Ma et al. 2019 [21] | Longshengzhi Capsule | 18 weeks | 10 weeks | | | Artery sections: APA↓; MOMA2↓; NCA↓CPA↑FCA↑SMC↑; TUNEL↓; ABCA1↑ ABCG1↑.  Liver sections: lipid droplets↓ Liver TG↓; FA oxidation↑ FA synyhesis↓ SREBP1↓; SREBP2↓ LDLR↑ HMGCS↓; DGAT1↓ ATGL↑ MTTP↓ APOC2↑; CCR2↓ IL-6↓ MCP-1↓ TNF-α↓; CD68↓ MOMA2↓.  Serum TNF-α↓ | | | | | | Reducing AS by reducing macrophage/foam cell accumulation, maintaining the integrity of arterial wall, ameliorating hepatic lipid metabolism and inhibiting inflammation. | | | | |
| HFD apoE-/- mice | Lu et al. 2019 [23] | Shexiang Baoxin Pill | 20 weeks | 20 weeks | | | | | APA↓; SOD↑ CAT↑ GSH↑ MDA↓ H2O2↓ and MPO↓; MCP-1↓ IFN-γ↓ IL-17A↓ IL-10 ↑ TGF-β1↑; VCAM-1↓ ICAM-1↓ IL-6↓ IL-2↓; macrophages↓ ABCA1↑ ABCG1↑; p38↓ JNK↓ Mfn2↑ NF-κB↓ SR-A↓ LOX-1↓ LXRα↑ | | | | | | | Exerting anti-atherosclerotic effects via improving inflammation response and inhibiting lipid accumulation. | |
| HFD male apoE-/- mice with silastic collar implantation | Chen et al. 2018 [31] | Tongxinluo Capsule | 8 weeks | | | 8 weeks | | | | | | TUNEL↓Lc3b dots↑; APA↓; Vulnerable index↓ macrophage apoptosis↓ | | | | Improving autophagy via Beclin-1. | |
| Female apoE-/- mice combined partial ligation of the left common carotid artery and left renal artery | Shen et al. 2017 [33] | Xuezhikang | 0 | 8 weeks | | | | | APA↓; CD68↓ α-SMA↑ CPA↑ Vulnerable Phenotype↓; p-PERK↓ p-IRE1α↓ p-eIF2α↓ and BiP↓ CHOP↓ DHE staining↓; NCA↓ TUNEL↓ caspase-3↓; TNFα↓ MMP8↓ and MMP13↓ | | | | | | | Suppressing vulnerable plaque progression and rupture by mitigating lesional endoplasmic reticulum stress and inhibiting apoptosis and the NF-κB pro-inflammatory pathway. | |
| HFD male or female apoE-/- or LDLR-/- mice | Chen et al. 2014 [55] | Danhong Injection | 16 weeks/20 weeks | | 16 weeks/20 weeks | | | | | | Male apoE-/-:ABCA1↑TNF-α↓;  female apoE-/-: APA↓ LDL-C↓ HMGCR↓ LDLR↑ TNF-α↓;  male ldlr-/-: ABCA1↑ TNF-α↓  female ldlr-/-: ABCA1↑ APA↓ HMGCR↓TNF-α↓ | | | | | Inhibiting AS through amelioration of lipid profiles. | |
| HFD male LDLR-/- mice | Zhao et al. 2013 [58] | Naoxintong Capsule | 8 weeks | 8 weeks | | | | | TC↓TG↓; APA↓; CD68↓; DCs↓ CD40↓ CD86↓ CD80↓ plasma IL-12p70↓ | | | | | | | Protecting against AS through lipid-lowering and inhibiting DCs maturation. | |
| HFD LDLR-/- mice | Liu et al. 2019 [73] | Shexiang Baoxin Pill | 14 weeks | 14 weeks | | | | α-SMA↓ SM22α↓ OPN↓ | | | | | | Reversing the dedifferentiation of VSMCs | | | |
| HFD male Japanese rabbits with the silastic collar implantation around the right carotid artery | Zhai et al. 2019 [20] | Zhixiong Capsule | 12 days | 12 days | | | | | TC↓HDL-C↑TC/HDL-C ratio↓log(TG/HDL-C)↓; IL-4↑; APA↓IA↓IA/MA↓ | | | | | | | Preventing atherosclerotic plaque formation and intimal thickening. | |
| HFD male and female New Zealand rabbits with the silicone tube encapsulation of left carotid artery | Yin et al. 2018 [27] | Tongxinluo Capsule | 4 weeks | 4 weeks | | | | | | TC↓ TG↓ LDL-C↓; Serum MDA↓ SOD↑, and T-AOC↑; VEGF-A↓ VEGF-R2↓; Nuclear NF-κB↓ TNF-α↓ IL-6↓; Nuclear Nrf2↑ NQO1↑ | | | | | Reducing carotid adventitial VV angiogenesis and alleviating early AS lesions by inhibiting carotid inflammation and oxidative stress injury. | |  |
| HFD New Zealand rabbits with the silastic collar implantation around the right carotid artery | Lang et al. 2015 [46] | Tongxinluo Capsule | 4 weeks | 4 weeks | | | | | IT↓; CD34↓; Microvascular blood flow volume↓；VAGF↓ VEGFR-2↓ | | | | | | | Inhibiting VV proliferation. | |
| HFD male New Zealand rabbits with balloon injury in aorta. | Han et al. 2011 [60] | Dahuang Zhechong Pill | 60 days | 60 days | | | | | Serum: MDA↓SOD↑ NO↑  Aorta: MPO↓ VSMCs: PCNA↓ Bcl-2↓ | | | | | | | Inhibiting AS through anti-lipid peroxidation, protection of vascular endothelium, inhibition of VSMCs proliferation and promotion of VSMCs apoptosis | |
| HFD male Japanese rabbits with balloon injury | Li et al. 2006 [67] | Tongxinluo Capsule | 16 weeks | 16 weeks | | | | | ET↓ NO↑; IT↓; CPA↑; MMP-1↓, COX-2↓; Bcl-2↑; FasL↓; Macrophage↓ | | | | | | | Reducing endothelial injury and intima thickness, inhibiting apoptosis and stabilizing plaques. | |
| HFD male Japanese rabbits | Song et al. 2010 [62] | Tongxinluo Capsule | 14 weeks | 14 weeks | | | | | TC↓ LDL↓; PAI-1↓ VCAM-1↓ | | | | | | | Inhibiting AS related to the reduction of blood lipid and inflammation. | |
| HFD male Japanese rabbits | Cao et al. 2009 [64] | Tongxinluo Capsule | 14 weeks | 14 weeks | | | | | APA↓; MMP-3↓ MMP-9↓ PPARγ↑ | | | | | | | Inhibiting the expression of MMP-3 and MMP-9 and increasing the expression of PPARγ. | |
| HFD Japanese rabbits | Xie et al. 2006 [66] | Xuezhikang | 12 weeks | 12 weeks | | | | | APA↓; TC↓ HDL-C↑ TG↓ LDL-C↓; Serum NO↑ CRP↓ | | | | | | | Inhibiting AS related to the reduction of blood lipid and inflammation. | |
| HFD New Zealand rabbits | Zhong et al. 2013 [57] | Naoxintong Capsule | 12 weeks | 12 weeks | | | | | LDL-C↓ TC↓; Aorta: iNOS mRNA↓ NO↓ | | | | | | | Reducing iNOS expression in AS lesions | |
| HFD male New Zealand rabbits | Fu et al. 2009 [63] | Danhong Injection | 14 weeks | 14 weeks | | | | | TC↓ TG↓ LDL-C↓; MDA↓ iNOS↓ COX-2↓; APA↓ | | | | | | | Inhibiting AS related to the reduction of blood lipid, the inhibition of arterial wall inflammation and the regulation of oxidative stress level. | |
| HFD male New Zealand rabbits | Yu et al. 2006 [65] | Tongxinluo Capsule | 16 weeks | 16 weeks | | | | | APA↓; TC↓ LDL↓; Macrophage↓; LOX-1↓ | | | | | | | Inhibiting AS related to the reduction of blood lipid and LOX-1. | |
| HFD male New Zealand rabbits | Tian et al. 2004 [68] | Fufang Danshen Dropping Pill | 12 weeks | 12 weeks | | | | | TC↓ HDL-C↑ TG↓ LDL-C↓; IT↓ | | | | | | | Reducing the blood lipid. | |
| HFD male New Zealand rabbits | Chen et al. 2004 [69] | Fufang Danshen Dropping Pill | 12 weeks | 12 weeks | | | | | LO↓ APA↓; VCAM-1↓ | | | | | | | Inhibiting VCAM-1 expression. | |
| HFD New Zealand rabbits with balloon-induced abdominal aortic endothelial injury, undergoing plaques triggering by Chinese Russell viper venom | Chen et al. 2009 [71] | Tongxinluo Capsule | 20 weeks | 12 weeks | | | | | Serum TC↓ LDL-C↓ TG↓; MCP-1↓ hs-CRP↓ IL-8↓ IL-18↓ MMP-1↓ P-selectin↓; Ultrasonography measurements: IMT↓; corrected AII ↑ APA↓ EEMA↓; MCP-1↓ MMP-1↓ MMP-3↓ MMP-12↓ P-selectin↓; vulnerability index↓ α-SMCs↑ CPA↓ Lipid↓ RAM-11↓ | | | | | | | Enhancing the stability of vulnerable plaques via effects on lipid lowering and anti-inflammation. | |
| HFD New Zealand rabbits with balloon-induced abdominal aortic endothelial injury, undergoing plaques triggering by Chinese Russell viper venom | Zhang et al. 2009 [72] | Tongxinluo Capsule | 10 weeks | 8 weeks | | | | | Serum TC↓ LDL↓ TG↓ HDL↑; MCP-1↓ hs-CRP↓ sICAM-1↓ ox-LDL↓; Ultrasonography measurements: corrected AII ↑ APA↓ EEMA↓; MCP-1↓ MMP-1↓ MMP-3↓ MMP-12↓ P-selectin↓; vulnerability index↓ α-SMCs↑ CPA↓ Lipids↓ Macrophages↓ Fibrous Cap Thickness↓; LOX-1↓ MMP-1↓ MMP-3↓ TIMP-1↓ NF-κB↓ | | | | | | | Enhancing the stability of plaque and preventing plaque rupture via lipid lowering, anti-inflammation and anti-oxidation. | |
| HFD male SD rats with Vitamin D3 injection | Fu et al. 2017 [35] | Angong Niuhuang Pill | 17 weeks | 9 weeks | | | | | APA↓IT↓MT↓, the maximum platelet aggregation rates↓; serum TC↓ LDL-C↓ TC/HDL-C↓ LDL-C/HDL-C↓; MDA↓ hsCRP↓ LDH↓ cTnI↓; Myocardial fibers↓; Bax protein↓ Bcl-2↑ | | | | | | | Reducing AS due to its anti-platelet aggregation, lipid regulatory, antioxidant, anti-inflammatory and anti-apoptotic properties. | |
| HFD male SD rats with Vitamin D3 injection | Cheng et al. 2015 [48] | Yindanxinnaotong Soft Capsule | 9 weeks | 12 weeks | | | | | APA↓TC↓TG↓LDL-C↓; MDA↓SOD↑GSH↑GSH-px↑; NF-kB↓ IkB↑; IL-1β↓ CRP↓ TNF-α↓; NO↑ TXB2↓ | | | | | | | Relieving AS through regulating lipids, reducing lipid particle deposition in the endothelial layer of artery, enhancing antioxidant power, and repressing inflammation activity by inhibiting NF-κB signal pathway. | |
| HFD male SD rats with Vitamin D3 | Guo et al. 2014 [54] | Suxiaojiuxin Pill | 12 weeks | 12 weeks | | | | | TG↓ LDL↓ TC↓ HDL↑ | | | | | | | Reducing lipids. | |
| HFD male SD rats with Vitamin D3 injection | Li et al. 2011 [61] | Suxiaojiuxin Pill | 12 weeks | 12 weeks | | | | | Serum: MDA↓ SOD↑ ox-LDL↓;  PPAR γ↓; NF-κB↓ | | | | | | | Anti-inflammation and inhibiting of oxidative stress, | |
| HFD male SD mice with VitaminD3 | Zhai et al. 2020 [78] | Zhixiong Capsule | 18 weeks | 6 weeks | | | | | APA↓ IA/MA ratio↓ CPA↑ mineralization↓; Serum TC↓LDL↓HDL↑; Thoracic arteries IL-4↑IL-13↑MAPK1↓ MAPK14↓p53↑ | | | | | | | Inhibiting AS plaque progression related to the reduction of blood lipid, macrophage content and macrophage transformation. | |
| Male SD rats with the left carotid artery balloon injury | Yao et al. 2014 [51] | Tongxinluo Capsule | 0 | 2 weeks | | | | | Serum ET-1↓MCP-1↓sICAM-1↓NO↑; Artery: ICAM-1↓MCP-1↓; the neointimal thickening↓ | | | | | | | Improving endothelial function, attenuating neointimal formation, and reducing inflammation. | |
| HFD male SD rats | Zhou et al. 2018 [26] | Longxuetongluo Capsule | 4 weeks | 4 weeks | | | | | TC↓ HDL-C↑ LDL-C↓ TG↓; Serum ALT↓ AST↓ Serum MCP-1↓ ICAM-1↓ VCAM-1↓.  Histological sections of liver and aorta↓; Aortic histological sections: NF-κB↓ | | | | | | | Preventing AS and fatty liver by controlling lipid metabolism and anti-inflammation activity. | |
| HFD male Wistar rats with Vitamin D3 injection | Chen et al. 2016 [42] | Danlou Tablet | 4 weeks | 8 weeks | | | | | TC↓ TG↓ LDL-C↓; APA↓; IL-6↓ TNF-α↓ MCP-1↓ ox-LDL↓;LP-PLA2↓ sPLA2↓ | | | | | | | Inhibiting AS related to the reduction of blood lipid and inflammation. | |
| HFD male Wistar rats with Vitamin D3 injection | Zhu et al. 2013 [56] | Xuezhikang | 12 weeks | 12 weeks | | | | | TG↓ LDL-C↓; Aorta caveolin-1↓; MDA↓ SOD↑ and T-AOC↑; eNOS↑, plasma NOx↑, cGMP in erythrocyte plasma and aorta wall↑; EDI↓ blood viscosity↓ | | | | | | | Elevating eNOS/NO, improving hemorheology and inhibiting oxidative stress. | |
| HFD male Wistar rats with Vitamin D3 injection | Li et al. 2011 [59] | Xuezhikang | 12 weeks | 12 weeks | | | | | LDL-C↓ TC↓; APTT↑ PT↑ TT↑ Fibrinogen↓ tissue factor↓SOD↑ MDA↓ | | | | | | | Inhibiting the tissue factor expression and reducing oxidative stress. | |
| HFD male Wistar rats with Vitamin D3 injection | Miao et al. 2016 [40] | Danlou Tablet/Xuefu Zhuyu Granule | 4 weeks | 8 weeks | | | | | Danlou group: IT↓ TC↓ TG↓ LDL-C↓ PDGF↓ ERK1/2↓ pERK1/2↓.  Xuefu Zhuyu group: IT↓ TC↓ PDGF↓ ERK1/2↓ pERK1/2↓ | | | | | | | Reducing serum lipids and increasing PDGF, inhibiting ERK signal pathway activation and VSMC proliferation. | |
| HFD male Wistar rats with Vitamin D3 injection and balloon injury in aorta. | Zhu et al. 2016 [37] | Ginkgo Biloba Tablet | 60 days | 60 days | | | | | Blood glucose and calcium↓; TC↓ TG↓ LDL-C↓; LO↓ IT↓; SR-A↓; CRP↓ ICAM-1↓ VCAM-1↓ | | | | | | | Alleviating AS lesions by inhibiting inflammation and controlling lipid. | |
| Male Wistar rats with the silicone collar around the left carotid artery | Guan et al. 2015 [70] | Tongxinluo Capsule | 0 | 4 weeks | | | | | pERK1/2↑ nNOS↑; LO↓ | | | | | | | Improving the blood flow and attenuating the chronic vasoconstriction through activation of ERK1/2 signaling. | |
| Male C57BL/6 mice with the left common carotid artery ligation | Zhang et al. 2014 [49] | Tongxinluo Capsule | 0 | 21 days | | | | | IA/MA ratio↓IA↓; TNF-α↓IL-1β↓; miR-155↓ | | | | | | | Inhibiting the vascular inflammatory response and neointimal hyperplasia. | |

AAA, abdominal aortic aneurysms; ABCA1, ATP binding cassette transporter A1; ABCG1, ATP binding cassette transporter G1; ACAT, acyl coenzyme A: cholesterol acyltransferase; ANGPT-1, angiopoietin-1; APOA1, apolipoprotein A I; APOB, apolipoprotein B; AS, atherosclerosis; APA, atherosclerotic plaque area; AI, atherosclerosis index values; AII, acoustic intensities; Akt, serine/threonine kinase; AMPK, adenosine monophosphate-activated protein kinase; CHOP, CCAAT-enhancer-binding protein homologous protein; CPA, collagen positive area; DC, dendritic cell; EDI, erythrocyte deformation index; EEMA, external elastic membrane area; FAS, fatty acid synthase; FBG, fasting blood glucose; FCA, fibrous cap area; FCT, fibrous cap thickness; FFA, free fatty acid; FINS, fasting insulin; GLUT-4, glucose transporter-4; GSH-PX, glutathione peroxidase; GSH, glutathione; HHcy, hyper-homocysteinemia; HMGCR, HMG-CoA reductase; HMGCS, HMG-CoA synthase; HO-1, heme oxygenase-1; hs-CRP, high-sensitivity C-reactive protein; ICAM-1, intercellular adhesion molecules-1; IL-6, interleukin-6; IR: insulin resistance; IA, intimal area; IT, intima thickness; IMT, intima–media thickness; LA, luminal area; LCAT, lecithin-cholesterol acyltransferase; LDH, lactate dehydrogenase; LOX-1, lectin-like oxidized low-density-lipoprotein receptor-1; LO, luminal occlusion; LP-PLA2, lipoprotein-associated phospholipase A2; LXRα, liver X receptor α; MA, medial area; MDA, malondialdehyde; NADPH, nicotinamide adenine dinucleotide phosphate; NQO1, NADPH quinone oxidoreductase 1; NCA, necrotic core area; NEFA, non-esterified fatty acid; NF-𝜅B, nuclear factor-kappa B; Nrf2, nuclear factor erythroid-2-related factor 2; OPN, osteopontin; ox-LDL, oxidized low-density lipoprotein; PAI-1, plasminogen activator inhibitor 1; PI3K, phosphatidylinositol-3-kinases; PPARγ, peroxisome proliferator-activated receptor γ; RCT, reverse cholesterol transport; SD, Sprague-Dawley; α-SMA, alpha smooth muscle actin; SM22α, smooth muscle 22 alpha; SR-B1, scavenger receptor class B type 1; SR-A1, scavenger receptor class A type 1; SOD, superoxide dismutase; sPLA2, secretory phospholipase A2; TNF-𝛼, tumor necrosis factor-𝛼; VV, vasa vasorum; VEGF-A, vascular endothelial growth factor A; VCAM-1, vascular cell adhesion molecule 1; VSMCs, vascular smooth muscle cells; VEGF, vascular endothelial growth factor.
