# Supplementary material for: Atheroprotective Effects and Mechanisms of Postmarketing Chinese Patent Formulas in Atherosclerosis Models: A Systematic Review
Source: Evid Based Complement Alternat Med. 2021 Nov 27;2021:4010607. doi: 10.1155/2021/4010607 (PMC8643251; doi:10.1155/2021/4010607)
Supplement: Supplementary Materials — Table S1. Classification of the postmarketing Chinese patent formulas in vivo. Table S2. Classification of the AS models in vivo. Table S3. Details of herbal drugs of the included formulas. Table S4. The bias of included animal studies according to SYRCLE's ROB tool. [file 4010607.f1.zip › 4010607.f1/Supplementary Table S3 (1).docx]

Supplementary *Table 3*: Details of herbal drugs of the included formulas

| Drug name | Approval numbers by NMPA | Pharmaceutical company | Main Chinese herbs |
| --- | --- | --- | --- |
| Danhong injection | Z20026866 | Danhong Pharmaceutical Co. Ltd  (Shandong, China) | Salviae Miltiorrhizae Radix Et Rhizoma, Carthami Flos |
| Zhixiong capsule | Z20163104 | Qianhui Co. Ltd (Taiyuan, China). | Hirudo, Chuanxiong Rhizoma, Salviae Miltiorrhizae Radix Et Rhizoma, Puerariae Lobatae Radix, Leonuri Herba |
| Longshengzhi capsule | Z20010059 | Buchang Pharmaceutical Co, Ltd (Shan’xi, China) | Astragali Radix, Hirudo, Chuanxiong Rhizoma, Angelicae Sinensis Radix, Carthami Flos, Persicae Semen, Paeoniae Radix Rubra, Aucklandiae Radix, Acori Tatarinowii Rhizoma, Pheretima, Taxilli Herba, Acanthopanax Extract |
| Tongxinluo capsule | Z19980015 | Shijiazhuang Yiling Pharmaceutical Co. Ltd (Shijiazhuang, Hebei, China) | Ginseng Radix Et Rhizoma, Hirudo, Scorpio, Paeoniae Radix Rubra, Cicadae Periostracum, Eupolyphaga Steleophaga, Scolopendra, Santali Albi Lignum, Dalbergiae Odoriferae Lignum, Olibanum, Ziziphi Spinosae Semen, Borneolum Syntheticum |
| Shexiang Baoxin pill | Z31020068 | Shanghai Hutchison Pharmaceuticals | Artificial Moschus, Total Ginsenoside Ginseng Root, Bovis Calculus Artifactus, Cinnamomi Cortex, Styrax, BufonisVenenum, Borneolum Syntheticum |
| Danlou tablet | Z20050244 | Jilin Connell Medicine Co. Ltd (Jilin, China) | Trichosanthis Pericarpium, Allii Macrostemonis Bulbus, Puerariae Lobatae Radix, Chuanxiong Rhizoma, Salviae Miltiorrhizae Radix Et Rhizoma, Paeoniae Radix Rubra, Alismatis Rhizoma, Astragali Radix, Drynariae Rhizoma, Curcumae Radix |
| Angong Niuhuang pill | Z44020047 | Guangzhou BaiyunshanZhongyi Pharmaceutical Co. Ltd  (Guangzhou, China) | Bovis Calculus Sativus, Powerdered Buffalo Horn Extract, Artificial Moschus, Margarita, Cinnabaris, Realgar, Coptidis Rhizoma, Scutellariae Radix, Gardeniae Fructus, Curcumae Radix, Borneolum Syntheticum |
| Longxuetongluo capsule | Z20130012 | JangsuKanion Pharmaceutical Co. Ltd (Jiangsu, China) | Phenolic extract of Draconis Sanguis |
| Longhu Rendan | Z20025168 | Shanghai Zhonghua pharmaceutical Co. Ltd | L-menthol, Borneolum Syntheticum, Caryophylli Flos, Amomi Fructus, Anisi Stellati Fructus, Cinnamomi Cortex, Piperis Fructus, Aucklandiae Radix, Zingiberis Rhizoma, Catechu, Glycyrrhizae Radix Et Rhizoma, [glutinous](javascript:;) [rice](javascript:;) [flour](javascript:;), [sodium](javascript:;) [benzoate](javascript:;), [red](javascript:;) [iron](javascript:;) [oxide](javascript:;), [dextrin](javascript:;), TalciPulvis, [methylsilicone](javascript:;) [oil](javascript:;), [graphite](javascript:;) [powder](javascript:;), c[ethyl](javascript:;) [alcohol](javascript:;) |
| Naoxintong pill/capsule | Z20090527/ Z20025001 | Buchang Co. (Shandong, China) or Xianyang Buchang Pharmaceutical Co. Ltd (Shan’xi, China) | Astragali Radix, Paeoniae Radix Rubra, Salviae Miltiorrhizae Radix Et Rhizoma, Angelicae Sinensis Radix, Chuanxiong Rhizoma, Persicae Semen, Carthami Flos, Olibanum, Myrrha, Spatholobi Caulis, Achyranthis Bidentatae Radix, Cinnamomi Ramulus, Mori Ramulus, Pheretima, Scorpio, Hirudo |
| Di'aoXinxuekang capsule | Z10910051 | Chengdu Diao Pharmaceutical Group Co. Ltd (Chengdu, China) | Extract from the rhizome of Dioscoreanipponica Makino and Dioscoreapanthaica Prain et Burkill |
| Xuezhikang | Z20080068 | WBL Peking University Biotech Co. Ltd (Beijing, China) | Extract from red yeast rice |
| Qishenyiqi pill | Z20030139 | Tasly Pharmaceutical Co. Ltd (Tianjin, China) | Astragali Radix, Salviae Miltiorrhizae Radix Et Rhizoma, Notoginseng Radix Et Rhizoma, Dalbergiae Odoriferae oil |
| Ginkgo Biloba tablet | Z20027949 | Yangzijiang Pharmaceutical Co. Ltd | Ginkgo leaves extract |
| Xuefu Zhuyu granule | Z20163068 | Zhejiang Conba Pharmaceutical Co. Ltd | Persicae Semen, Carthami Flos, Angelicae Sinensis Radix, Chuanxiong Rhizoma, Rehmanniae Radix, Paeoniae Radix Rubra, Achyranthis Bidentatae Radix, Bupleuri Radix, Aurantii Fructus, Platycodonis Radix, Glycyrrhizae Radix Et Rhizoma |
| Shexiang Tongxin Dropping pill | Z20080018 | Inner Mongolia Conba Pharmaceutical Co. Ltd | Artificial Moschus, total ginsenoside of ginseng stems and leaves, Bufonis Venenum, Salviae Miltiorrhizae Radix Et Rhizoma, Bovis Calculus Artifactus, [bear](javascript:;) [gall](javascript:;) [powder](javascript:;), Borneolum Syntheticum |
| Compound Chuanxiong capsule | Z20000035 | Shandong Phoenix Pharmaceutical Co. Ltd (Shandong, China) | Chuanxiong Rhizoma, Angelicae Pubescentis Radix, Chaenomelis Fructus |
| Yindanxinnaotong soft capsule | Z20027144 | Guizhou Bailing Pharmaceutical Co. Ltd | Ginkgo Folium, Salviae Miltiorrhizae Radix Et Rhizoma, Erigerontis Herba, [Gynostemma](javascript:;) [Pentaphylla](javascript:;), Crataegi Fructus, AlliiSativi Bulbus, Notoginseng Radix Et Rhizoma, l-Borneolum |
| Dahuang Zhechong pill | Z61021089 | Xi’an Zhengda Pharmaceutical Co. Ltd (Xi’an, Shanxi, China) | Rhei Radix Et Rhizoma, Eupolyphaga Steleophaga, Hirudo, gadfly, grub, Toxicodendri Resina, Persicae Semen, Armeniacae Semen Amarum, Scutellariae Radix, Rehmanniae Radix, Paeoniae Radix Alba, Glycyrrhizae Radix Et Rhizoma |
| Fufang Danshen dropping pill | Z10950111 | Tasly Pharmaceutical Co. Ltd (Tianjin, China) | Salviae Miltiorrhizae Radix Et Rhizoma, Notoginseng Radix Et Rhizoma, Borneolum Syntheticum |
| Suxiaojiuxin pill | Z12020025 | Tianjin Zhongxin Pharmaceutical Co. Ltd | Chuanxiong Rhizoma, Borneolum Syntheticum |
| Xuezhitong capsule | Z10970076 | Dongfang Pharmaceutical Co. Ltd (Jilin, China) | Allii Macrostemonis Bulbus |
| Guanxinshutong capsule | Z20020055 | Buchang Pharmaceutical Co. Ltd (Shan’xi, China) | Choerospondiatis Fructus, Salviae Miltiorrhizae Radix Et Rhizoma, Caryophylli Flos, Borneolum Syntheticum, Bamusaeconcretiosilicea |

Co., Ltd, limited company; NMPA, National Medical Products Administration
