# Supplementary material for: Atheroprotective Effects and Mechanisms of Postmarketing Chinese Patent Formulas in Atherosclerosis Models: A Systematic Review
Source: Evid Based Complement Alternat Med. 2021 Nov 27;2021:4010607. doi: 10.1155/2021/4010607 (PMC8643251; doi:10.1155/2021/4010607)
Supplement: Supplementary Materials — Table S1. Classification of the postmarketing Chinese patent formulas in vivo. Table S2. Classification of the AS models in vivo. Table S3. Details of herbal drugs of the included formulas. Table S4. The bias of included animal studies according to SYRCLE's ROB tool. [file 4010607.f1.zip › 4010607.f1/Supplementary Table S4.docx]

Supplementary *Table S4*: The bias of included animal studies according to SYRCLE’s ROB tool.

| Ref. | Sequence generation (Selection bias) | Baseline characteristics (Selection bias) | Allocation concealment (Selection bias) | Random housing (Performance bias) | Blinding (Performance bias) | Random outcome assessment (Detection bias) | Blinding (Detection bias) | Incomplete outcome data (Attrition bias) | Selective outcome reporting (Reporting bias) | Other sources of bias |
| --- | --- | --- | --- | --- | --- | --- | --- | --- | --- | --- |
| Zhou et al., 2019 | U | U | U | U | U | U | U | H | L | L |
| Zhai et al., 2019 | U | U | U | L | U | U | U | H | L | L |
| Ma et al., 2019 | U | U | U | U | U | U | U | H | L | L |
| Ma et al., 2019 | U | U | U | U | U | U | U | U | L | L |
| Lu et al., 2019 | U | U | U | L | U | U | U | H | L | L |
| Hao et al., 2019 | H | U | U | U | U | U | U | U | L | L |
| Chai et al., 2019 | U | U | U | U | U | U | U | U | L | L |
| Zhou et al., 2018 | U | U | U | L | U | U | U | H | L | L |
| Yin et al., 2018 | U | U | U | L | U | U | U | U | L | L |
| Yan et al., 2018 | U | U | U | U | U | U | U | U | L | L |
| Wang et al., 2018 | U | U | U | L | U | U | U | U | L | L |
| Qu et al., 2018 | U | U | U | L | U | U | U | U | L | L |
| Chen et al., 2018 | U | U | U | U | U | U | U | U | L | L |
| Yang et al., 2017 | U | U | U | U | U | U | U | H | L | L |
| Shen et al., 2017 | H | U | U | U | U | U | U | H | L | L |
| Peng et al., 2017 | H | U | U | L | U | U | U | H | L | L |
| Fu et al., 2017 | U | U | U | L | U | U | U | H | L | L |
| Dong et al., 2017 | U | U | U | L | U | U | U | H | L | L |
| Zhu et al., 2016 | U | U | U | U | U | U | U | L | L | L |
| Zheng et al., 2016 | U | U | U | L | U | U | U | U | L | L |
| Yang et al., 2016 | U | U | U | U | U | U | U | H | L | L |
| Miao et al., 2016 | L | U | U | U | U | U | U | L | L | L |
| Ma et al., 2016 | U | U | U | L | U | U | U | U | L | L |
| Chen et al., 2016 | L | U | U | U | U | U | U | L | L | L |
| Xiong et al., 2015 | L | U | U | U | U | U | U | L | L | L |
| Xiong et al., 2015 | U | U | U | U | U | U | U | L | L | L |
| Wu et al., 2015 | U | U | U | L | U | U | U | H | L | L |
| Lang et al., 2015 | U | U | U | L | U | U | U | H | L | L |
| Kang et al., 2015 | U | U | U | U | U | U | U | H | L | L |
| Cheng et al., 2015 | U | U | U | L | U | U | U | L | L | L |
| Zhang et al., 2014 | H | U | U | U | U | U | U | U | L | L |
| Zhang et al., 2014 | U | U | U | L | U | U | U | H | L | L |
| Yao et al., 2014 | U | U | U | U | U | U | U | H | L | L |
| Wang et al., 2014 | U | U | U | L | U | U | U | H | L | L |
| Liu et al., 2014 | U | U | U | U | U | U | U | H | L | L |
| Guo et al., 2014 | U | U | U | U | U | U | U | U | L | L |
| Chen et al., 2014 | U | U | U | U | U | U | U | H | L | L |
| Zhu et al., 2013 | U | U | U | U | U | U | U | H | L | L |
| Zhong et al., 2013 | U | U | U | U | U | U | U | U | L | L |
| Zhao et al., 2013 | U | U | U | U | U | U | U | L | L | L |
| Li et al., 2011 | H | U | U | L | U | U | U | L | L | L |
| Han et al., 2011 | U | U | U | U | U | U | U | H | L | L |
| Li et al., 2011 | U | U | U | U | U | U | U | H | L | L |
| Song et al., 2010 | U | U | U | U | U | U | U | L | L | L |
| Fu et al., 2009 | U | U | U | U | U | U | U | L | L | L |
| Cao et al., 2009 | U | U | U | U | U | U | U | U | L | L |
| Yu et al., 2006 | U | U | U | U | U | U | U | L | L | L |
| Xie et al., 2006 | L | U | U | U | U | U | U | L | L | L |
| Li et al., 2006 | U | U | U | U | U | U | U | L | L | L |
| Tian et al., 2004 | L | U | U | U | U | U | U | H | L | L |
| Chen et al., 2004 | U | U | U | U | U | U | U | L | L | L |
| Guan et al., 2015 | U | U | U | U | U | U | U | U | L | L |
| Chen et al., 2009 | U | U | U | U | U | U | U | U | L | L |
| Zhang et al., 2009 | U | U | U | U | U | U | U | U | L | L |
| Liu et al., 2019 | U | U | U | L | U | U | U | L | L | L |
| Meng et al., 2019 | U | U | U | U | U | U | U | H | L | L |
| Gao et al., 2020 | H | U | U | L | U | U | U | H | L | L |
| Lu et al., 2020 | H | U | U | U | U | U | U | H | L | L |
| Sun et al., 2020 | U | U | U | U | U | U | U | H | L | L |
| Zhai et al., 2020 | U | U | U | U | U | U | U | U | H | L |

H, high risk; L, low risk; U, unclear risk
